# Integrative analysis of somatic mutations and transcriptomic data to functionally stratify breast cancer patients

**DOI:** 10.1186/s12864-016-2902-0

**Published:** 2016-08-22

**Authors:** Jie Zhang, Zachary Abrams, Jeffrey D. Parvin, Kun Huang

**Affiliations:** Department of Biomedical Informatics, The Ohio State University, Columbus, OH 43210 USA

**Keywords:** Distance correlation, Breast cancer patient stratification, Functional analysis of somatic mutation, Integrative analysis

## Abstract

**Background:**

Somatic mutations can be used as potential biomarkers for subtyping and predicting outcomes for cancer patients. However, cancer patients often carry many somatic mutations, which do not always concentrate on specific genomic loci, suggesting that the mutations may affect common pathways or gene interaction networks instead of common genes. The challenge is thus to identify the functional relationships among the mutations using multi-modal data. We developed a novel approach for integrating patient somatic mutation, transcriptome and clinical data to mine underlying functional gene groups that can be used to stratify cancer patients into groups with different clinical outcomes. Specifically, we use distance correlation metric to mine the correlations between expression profiles of mutated genes from different patients.

**Results:**

With this approach, we were able to cluster patients based on the functional relationships between the affected genes using their expression profiles, and to visualize the results using multi-dimensional scaling. Interestingly, we identified a stable subgroup of breast cancer patients that are highly enriched with ER-negative and triple-negative subtypes, and the somatic mutation genes they harbor were capable of acting as potential biomarkers to predict patient survival in several different breast cancer datasets, especially in ER-negative cohorts which has lacked reliable biomarkers.

**Conclusions:**

Our method provides a novel and promising approach for integrating genotyping and gene expression data in patient stratification in complex diseases.

**Electronic supplementary material:**

The online version of this article (doi:10.1186/s12864-016-2902-0) contains supplementary material, which is available to authorized users.

## Background

The initiation, development, and metastasis of cancers are complicated processes involving multi-cell, multi-tissue interactions and communications. Most cancers confer heterogeneity among patients that lead to different clinical outcomes such as survival time and response to treatment. With recent rapid advancement in next generation sequencing (NGS) technologies and computing capacity for processing and storing large data, more and more human cancer genomes have been characterized in a systematic way, bringing great opportunities for researchers to carry out integrative analysis to identify potential molecular markers for stratifying patients into subtypes with different predicted clinical outcomes [1]. Currently The Cancer Genome Atlas (TCGA) project harbors comprehensive data ranging from genomic sequences, genetic variants, transcriptomic and proteomic data to clinical data for multiple types of human cancer tissues as well as normal tissues. It is a great source for scientists to integrate data from different levels and mine the buried interaction among them, which will shed light on the understanding of cancer subtyping, prognosis as well as the cancer initiation and development [2–4].

In TCGA database, we often observe patients with a lot of somatic mutations that can significantly alter corresponding protein structures or functions of the genes they reside on (we named the affected gene as significantly mutated gene, or SMG). SMGs are the results of splice-site-change, nonsense, non-stop or frame-shift mutations. The prevalence of SMGs in almost all cancer types let us postulate that they may be potentially used as signatures for subtyping and outcome prediction, or as starting point to elucidate the tumorigenesis process. However, there is a big challenge in using SMGs for cancer patient stratification — the overlaps between the SMGs from different patients are usually small and the lists are usually not converging to common pathways [1, 5]. For instance, the breast cancer (BRCA) project in TCGA has identified three commonly mutated genes TP53, GATA3, and PI3KC but every patient has a much larger number of somatic mutations which cannot be easily summarized and compared even at the pathway level [1]. Therefore, it is of great interest in identifying the potential relationships between the mutated genes from different patients.

In this paper, instead of directly working on the gene lists, we propose to examine the functional relationships of the SMGs between different patients based on functional genomics data. One of such functional measurements is gene expression profile obtained from microarray or RNA-seq experiments, which has already been curated in TCGA. Specifically, given two sets of SMGs from two patients, we develop a method to establish the relationship between them based on expression profiles of the two gene lists.

Given a list of genes with their expression profiless measured in a cohort of patients, one way to characterize their roles is to examine how these genes lead to separation of the patients. In other words, we can establish a “patient network” using the difference of the expression levels of the genes as distance metric. Then given two gene lists, we can compare the similarity between the patient networks established by each of the lists. The similarity will provide pivotal information on the similarity between the roles of these two gene lists among the patients.

Mathematically, such similarity between patient networks can be computed using a recently developed metric called distance correlation [6]. Therefore in this paper, we develop a workflow for establishing the functional similarity among SMGs from different patients based on distance correlation. Our goal is trying to reveal the yet unknown links between different SMG, which indicate their functional relationships in the context of human gene interaction network, and use this relationship to stratify patients with different subtypes. While we demonstrate our approach using a breast cancer study, our method provides a novel promising approach of integrating genotype and gene expression data in patient stratification in complex diseases.

## Methods

**Fig. 1:**
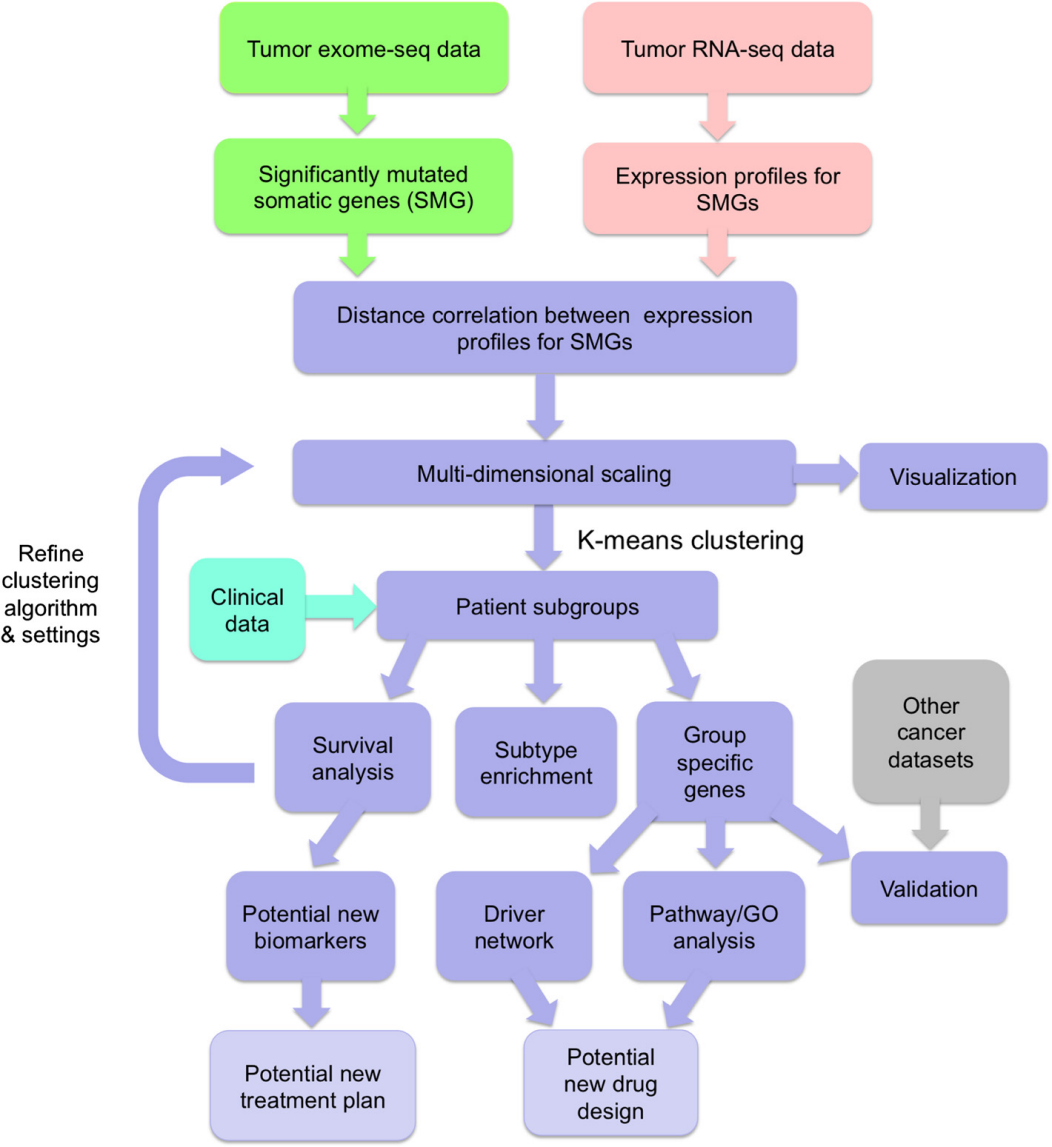
Workflow of identifying functional gene relationships using variants and transcriptomic data

In this paper, we obtained whole genome exome-seq data (WES) from TCGA for the patients with breast cancers and derived the SMG list for each patient. The list of SMGs from each patient were used as features for this patient. We then computed distance correlation of every pair of SMG lists to obtain the functional relationships between the affected genes in different patients based on the gene expression profiles. The process yielded the distance correlation matrix across the patients. Then we visualized the patients by multi-dimensional scaling, and further clustered the patients into different groups. Our workflow is summarized in Fig. 1.

The key component in this workflow is to compute the distance correlation between a pair of gene lists (in this case, expression profiles of two SMG lists from two patients). The intuition behind distance correlation can be considered as following: A gene list can be used to cluster the patient cohort of a heterogeneous disease, generating a clustering result. Two different gene lists will generate two results, and the results may be similar if the two gene lists play similar functional roles in the disease phenotype. The distance correlation measures the similarity of the two results.

In our case, we used the gene expression data (RNA-seq) of the entire cohort to compute the distance correlation, although theoretically, any gene expression dataset of a cohort with similar disease diversity can be used, and from a more general point of view, any type of data which present deep enough functional relationship among genes, even on normal people, can be used.

After we obtained the distance correlation matrix of any two SMG lists in the context of gene expression, which represents the functional relationship of any two sets of SMGs in the breast cancer disease gene expression, we use this matrix to cluster the entire breast cancer cohort, and the results should show a group of patients grouped by their common underlying perturbation resulted from seemingly different SMG lists.

### Datasets

The Cancer Genome Atlas (TCGA http://www.cancergenome.nih.gov) level-3 breast cancer patients’ somatic mutation derived from WES and RNA-seq data were downloaded from TCGA data portal in July, 2013. Among all 876 available patients at the time of download, 445 have matching SMG and RNA-seq data. The data from these patients were chosen for further analysis. 83 normal breast sample RNA-seq level 3 data were also obtained from TCGA.

### SMG selection

Somatic mutations derived from WES of the TCGA breast cancer patients were screened for significant mutation genes (SMG). SMG was defined as genes with frame-shift Indels, splice site change, non-stop mutation, or nonsense mutation. The mutation of mismatch, silent, RNA and in-frame indel were not included in SMG. For a specific group of patients, the number of SMG refers to the union of SMGs in that group of patients. For all the patients we analyzed in this study, their corresponding SMGs were listed in Additional file 1: Table S2.

### Computing distance correlation

Distance correlation is a recently developed metric with two advantages [6]. First, it can be used to calculate the “correlation” between two matrices instead of just two vectors. Essentially it calculates the similarity of effects of two “feature sets” on separating the same set of samples. Secondly, unlike Pearson correlation that is based on a linear model, it can respond to nonlinear relationships. These properties make it a good candidate for our purpose when comparing relationships between two gene lists.

In this project, the distance correlation was computed using Matlab as described in [6]. Given two lists of SMGs ${\mathit{\mathsf{g}}}_a$ and ${\mathit{\mathsf{g}}}_b$ with *n*_*a*_ and *n*_*b*_ genes respectively, we first extract their gene expression matrices across *N* patients as

$$ {E}^a=\left[\begin{array}{ccc}\hfill {e}_1^a\hfill & \hfill \cdots \hfill & \hfill {e}_N^a\hfill \end{array}\right]\in {\mathrm{\Re}}^{n_a\times N}\mathrm{and}\kern0.5em {E}^b=\left[{e}_1^b\kern1em \cdots \kern1em {e}_N^b\right]\in {\mathrm{\Re}}^{n_b\times N}, $$

where *e*_*j*_^*i*^ (*i* ∈ {*a*, *b*}, *j* ∈ {1, 2, …, *N*}) are *n*_*i*_- dimensional column vectors representing the expression profiles for the j-th patient over the i-th SMG list. The distance matrices among the patients for the two sets of SMGs can be calculated as

$$ {D}^a=\left[{d}_{jk}^a\right]\in {\mathrm{\Re}}^{N\times N}\mathrm{and}\kern0.5em {D}^b=\left[{d}_{jk}^b\right]\in {\mathrm{\Re}}^{N\times N} $$

with *d*_*jk*_^*i*^ = ‖*e*_*j*_^*i*^ − *e*_*k*_^*i*^‖, *i* ∈ {*a*, *b*}, *j*, *k* = 1, 2, …, *N*. Let $\overline{d_{j,\cdot}^i}$ and $\overline{d_{\cdot, k}^i}$ be the average of the j-th row and k-th column for the matrix *D*^*i*^ (*i* ∈ {*a*, *b*}) respectively. Also set $\overline{d_{\cdot, \cdot}^i}$ be the grand average of all entries of *D*^*i*^ (*i* ∈ {*a*, *b*}). Then set the centralized distance matrices to be

$$ \overline{D^i}=\left[\overline{d_{jk}^i}\right]=\left[{d}_{jk}^i-\overline{d_{j,\cdot}^i}-\overline{d_{\cdot, k}^i}+\overline{d_{j\cdot k}^i}\right]\in {\mathrm{\Re}}^{N\times N}\mathrm{with}\ i\ \in \left\{a,b\right\}. $$

Then the *distance covariance* between two distance matrices can be computed as

$$ dCov\left({E}^a,\ {E}^b\right)=\frac{1}{N^2}{\displaystyle {\sum}_{j,k=1}^N}\overline{d_{jk}^a}\cdot \overline{d_{jk}^b,} $$

and the ***distance correlation*** is defined as

$$ dCor\left({E}^a,\ {E}^b\right)=\frac{dCov\left({E}^a,{E}^b\right)}{\sqrt{dCov\left({E}^a,{E}^a\right)\cdot dCov\left({E}^b,\ {E}^b\right)}}. $$

For the 445 SMG lists obtained from the 445 patients, we compute the *distance correlation matrix*

$$ {D}_{dCor}=\left[ dCor\left({E}^i,{E}^j\right)\right]\in {\mathrm{\Re}}^{455\times 455},i,j=1,2,\dots, 445. $$

### Multidimensional scaling and clustering

In order to visualize the distribution of the patients with the proximity measurements defined by the distance correlation matrix, we applied multidimensional scaling (MDS) to embed the data points (each point represents a patient) in 3D space. Specifically we used Matlab function *cmdscale()* with its default settings. The distance correlation matrix was first transformed to a dissimilarity matrix (using 1 − *D*_*dCor*_) before MDS. K-means clustering was performed upon the patients using data using the same dissimilarity matrix. It was carried out using Matlab k-means function with default square-Euclidean distance and replicates of 50, *K* = 3 or 5.

### Jaccard index computing

SMGs for every pair of patients in TCGA BRCA cohort were used to calculate the similarity between the two SMG lists using Jaccard index (*J*), which is defined as:

$$ \boldsymbol{J}=\frac{\left|\boldsymbol{A}\ {\displaystyle \cap}\boldsymbol{B}\right|}{\left|\boldsymbol{A}\ {\displaystyle \cup}\boldsymbol{B}\right|}, $$

where A and B are the two groups of SMGs from any pair of patients in the TCGA BRCA cohort. A∩B is the set of overlapping genes within the two SMG groups A and B, and A∪B is the union of these two groups.

### Survival analysis

For validation, NCBI GEO breast cancer dataset GSE1456 (containing 318 patients of mixed types) [7] as well as Netherlands Kanker Instituut (NKI) NKI-295 dataset (containing 295 patients of mixed types) were used [8]. These microarray datasets (and their specific subtypes) contain gene expression data and matching survival time (years) that are needed for  survival analysis. Log-rank test was performed to determine the significance of difference in survival time between two patient groups and Kaplan-Meier curves were plotted.

### Pathway analysis and gene query in TCGA database

Ingenuity Pathway Analysis (IPA) was used to analyze enriched biological functions and pathways in the identified SMGs. The prevalence of SMGs on other cancer types in TCGA database was generated using the cBioPortal online tools (http://www.cbioportal.org) [9].

## Results

**Fig. 2:**
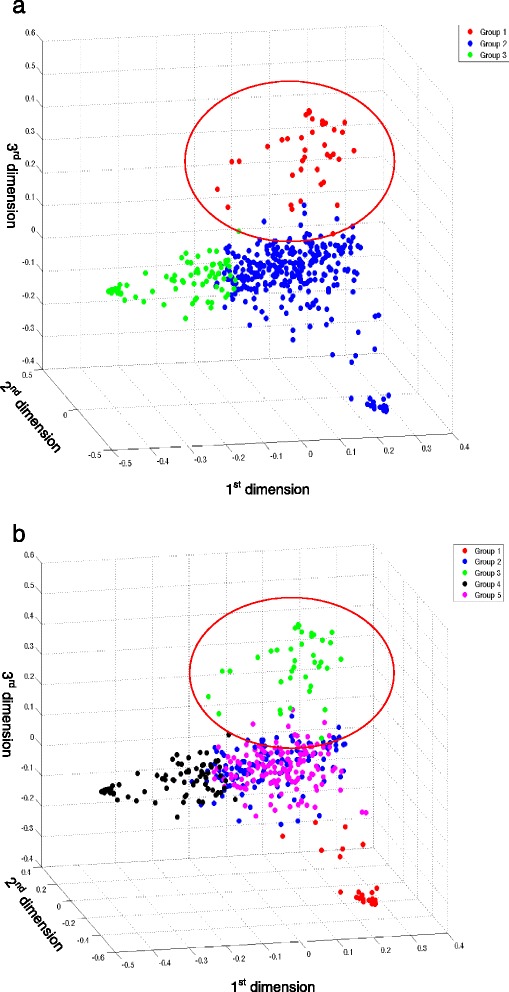
K-means clustering on the embedded patients , revealing a subtype of breast cancer patients enriched with triple-negative patients. **a**: *K* = 3, Red: Group 1, Blue: Group 2, Green: Group 3. **b**: *K* = 5, Group 2 from panel A was further clustered into three groups (blue, magenta and red)

We applied the above described workflow to analyze 445 breast cancer patients with matching SMG and RNA-seq data from TCGA. The distance correlation matrix was calculated and transformed. After MDS, the patients were imbedded into a 3D space for visualization, as shown in Fig. 2, with each point representing a patient.

When the patients were clustered using the K-means clustering algorithm, we observed a distinctive group of patients as highlighted by the red circle in Fig. 2. The number of clusters is tested by checking the silhouette values and plots for different choice of K. The silhouette value reaches its high peak at *K* = 5 (data not shown) but this group is stable even when the number of clusters changed (e.g., *K* = 3 vs. 5). In addition, we inspected the silhouette plots and found that the clusters are more separated when *K* = 3. Thus we use *K* = 3 for most the rest analysis.

**Fig. 3:**
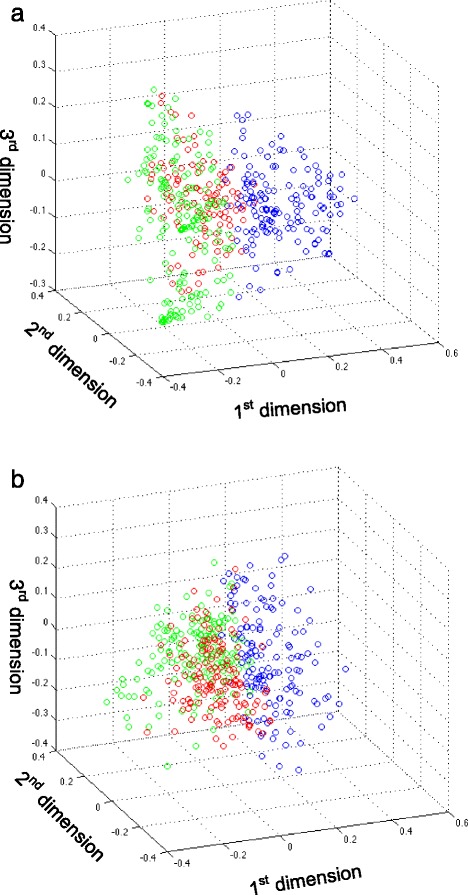
The results of the distance correlation workflow on control data. **a**: Applying the workflow using normal breast gene expression data. The three groups from K-means clustering do not enrich specific subtypes of breast cancers. **b**: Applying the workflow on randomly selected “pseudo-SMGs”. No subtype enriched patient cluster can be observed

In order to test if the clustering of patients can be achieved using other methods or could be an artifact, we carried out three tests. First, we directly used the SMGs as features for the patients and the similarity among the patients were established by calculating the Jaccard indicies between every pair of patients. Out of all the 98,790 patient pairs, 96.2 % are zeros, which means they do not share any common genes. Thus using SMGs cannot effectively separate the patients. Secondly, we tested if using non-cancer gene expression data can lead to the same observation. As shown in Fig. 3a, there is no clear separation among the patients and the clusters obtained from K-means algorithm do not have any enrichment of specific subtypes of breast cancers when we used 83 normal breast tissue samples RNA-seq data instead of breast cancer data. Finally, we tested randomly selected “pseudo-SMGs” for the patients. Basically for each patient, we randomly select the same number of genes as her SMGs, applying the same workflow and the result is shown in Fig. 3b. Similar results are observed as in Fig. 3a.

In order to gain insight on this distinctive group of patients, we examined the status of the known molecular markers for breast cancers, namely estrogen receptor (ER), progesterone receptor (PR), and HER2. Statistical analysis revealed that this group is significantly enriched with ER-negative, PR-negative, HER2-negative, or triple negative breast cancer (ER-, PR-, HER2- or TNBC) patients. Specifically, while it contains 41 patients consisting only 9.2 % of the total cohort, it includes 34 % of the total TNBC patients (Table 1). To examine if this group can be differentiated easily from the cohort using other genes, we repeated the process using randomly selected “pseudo SMGs” of the same sizes for every patient. The clustering result was not able to separate the patients into groups with such enrichment of the ER- or TNBC patients. Since both ER- and TNBC patients are known to have worse prognosis then the ER+ patients, our further analysis was focused on this specific group, and we refer it as the “Group 1” in the rest of the paper.

**Table 1:** Statistical tests on the patient subtypes enriched in each group from *K* = 3 clustering results. No statistic test was performed for HER2 (and TN) status, due to the fact that more than 25 % patients do not contain HER2 status

	Total	ER+	ER-	χ^2^ adj-P value	PR+	PR-	χ^2^ adj-P value	HER2+	HER2-	Triple -
Group1	41	14	26	0.00075	13	26	0.00658	8	23	16
Group2	304	233	68	0.700	205	95	0.488	55	158	28
Group3	100	85	12	0.0619	68	30	0.6476	15	53	5
Total (with sig mutation and matching RNAseq)	445	332	106		286	151		78	235	49
Total in TCGA	876	634	187		548	267		136	447	90

**Fig. 4:**
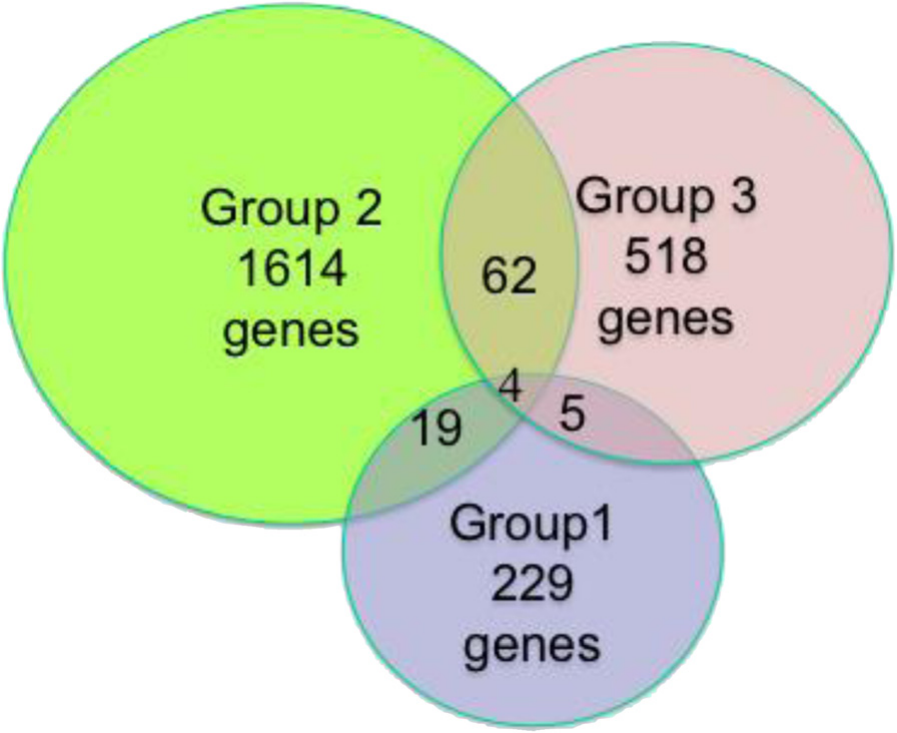
Venn diagram showing the genes shared/unique among the three groups from *K* = 3 clustering results

**Fig. 5:**
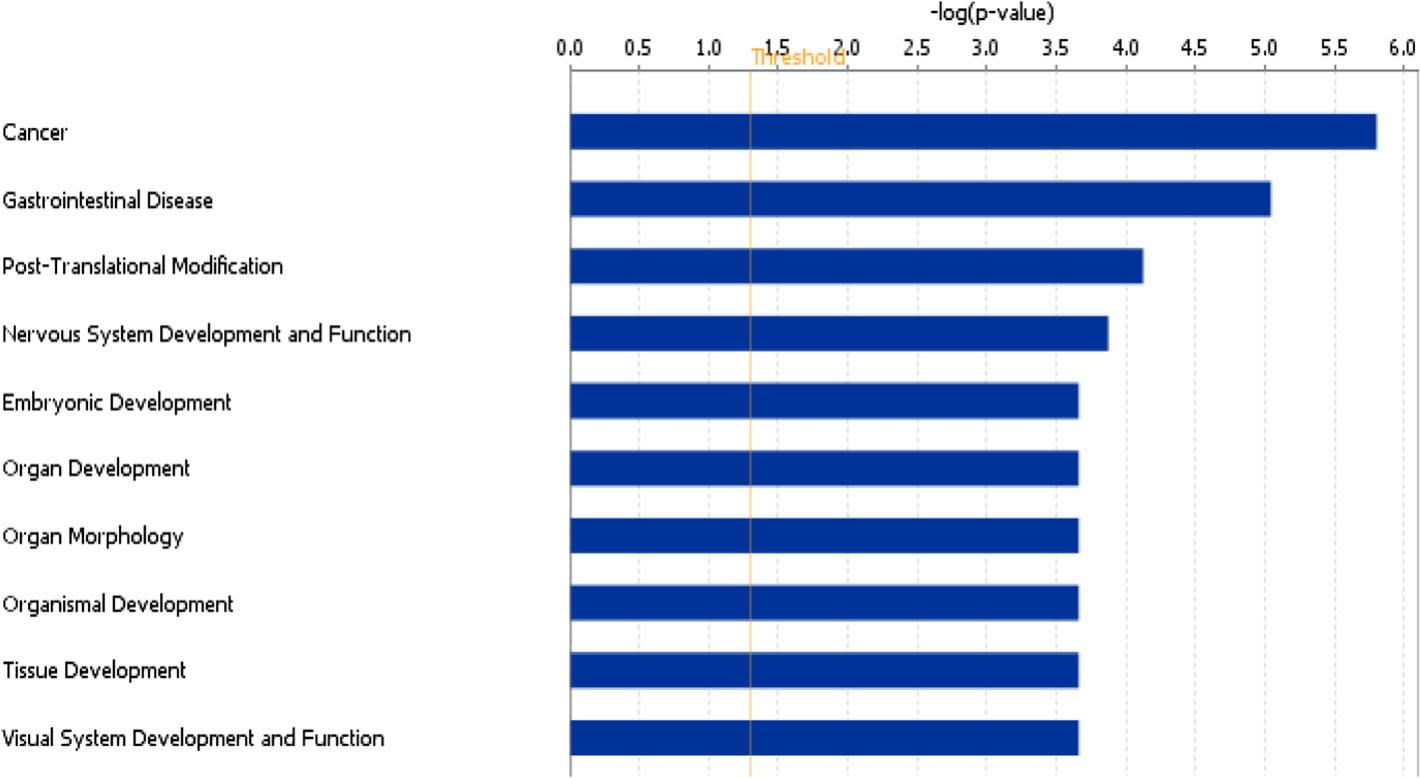
Pathway analysis showing the top 10 biological functions enriched in the genes specifically to Group 1 isolated from *K* = 3 clustering

Group 1 contains 201 SMGs that are specifically present in Group 1 patients (Fig. 4, Additional file 1: Table S1). Enrichment and pathway analysis using IPA showed that these SMGs are highly enriched with cancer-related genes, genes for embryonic development, cell morphology and organ development, indicating this group of genes are more involved in the early stage of cancer cell development and differentiation process (Fig. 5). Several upstream regulator drugs are found to regulate multiple genes in this group, among them, Ethinyl estradiol, an orally bioactive estrogen, regulates ABCB11, CCR7, CD97, CYP2D6, CYP7B1, SGK1, suggesting although being ER-negative, estrogen may still play a role in this group of patients; the drug, which is used to treat myelodsyplastic syndromes and acute myeloid leukemia, regulates BMP4, CCR7, MAGEC1, METAP2, MGMT, RARB, RARRES1, SGK1, SNRPN, and TGFBR2 [10, 11]. This may be a direction for future therapeutic research on this specific subtype of triple-negative breast cancer. Interestingly, the narcotic substance amphetamine regulates BMP4, DCC, SGK1, and TGFBR2.

**Fig. 6:**
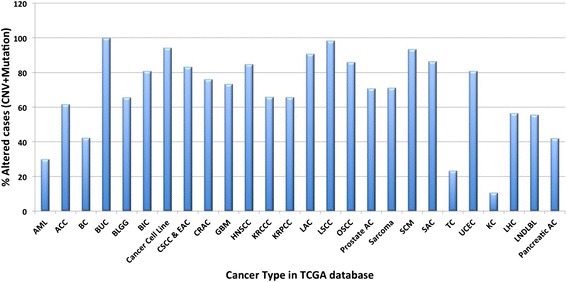
Group 1 specific genes are altered in multiple cancer types (TCGA data). AML: acute myeloid leukemia; ACC: adenoid cystic carcinoma; BC: bladder cancer; BUC: bladder urothelial carcinoma; BLGG: brain lower grade glioma; BIC: breast invasive carcinoma; CSCC &EAC: cervical squamous cell carcinoma & endocervical adenocarcinoma; GBM: glioblastoma multiforme; HNSCC: head & neck squamous cell carcinoma; KRCCC: kidney renal clear cell carcinoma; KRPCC: kidney renal papillary cell carcinoma; LAC: lung adenocarcinoma; LSCC: lung squamous cell carcinoma; OSCC: ovarian serous cystadenocarcinoma; Prostate AC: prostate adenocarcinoma; SCM: skin cutaneous melanoma; SAC: stomach adenocarcinoma; TC: thyroid carcinoma; UCEC: uterine corpus endometrial carcinoma; LHC: liver hepatic carcinoma; Pancreatic AC: pancreatic adenocarcinoma

In addition, analysis using cBioPortal shows that the group of 201 SMG genes is found frequently altered (mutated, or contain copy number variance) in almost all types of cancers available in TCGA database (Fig. 6).

**Fig. 7:**
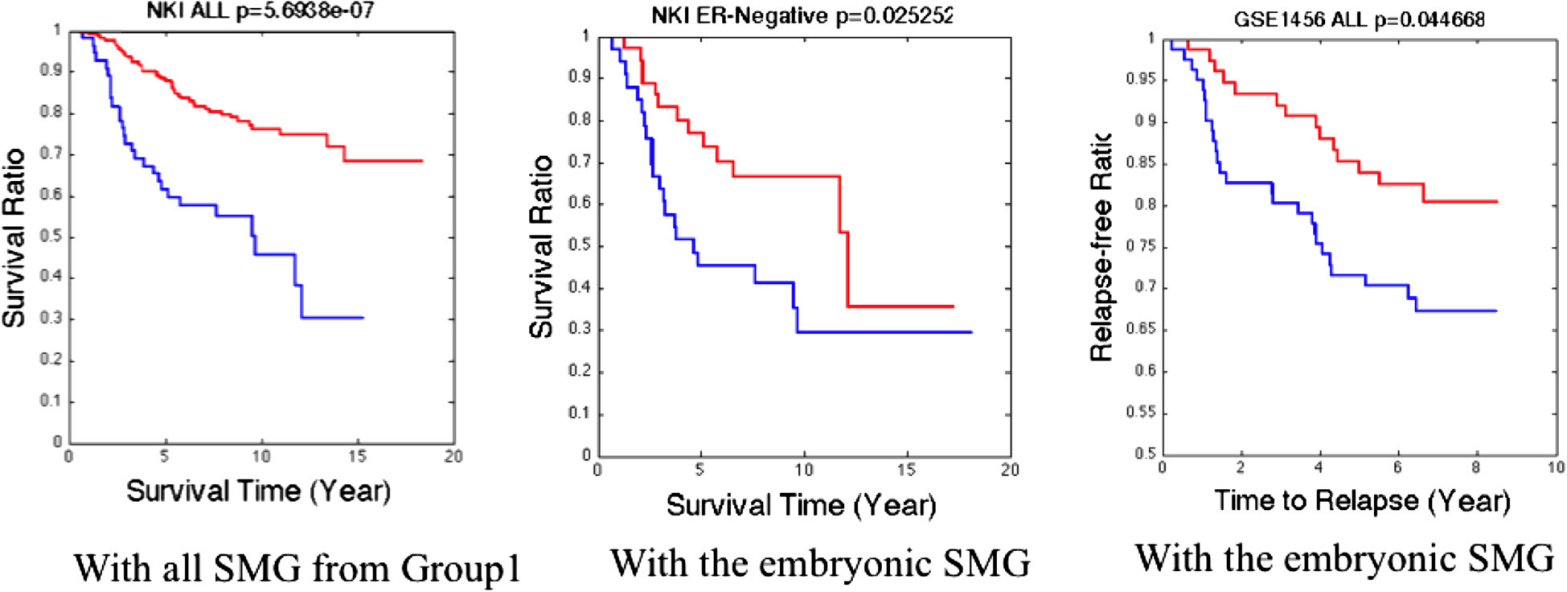
Survival analysis using the Group 1 specific genes and its subset on separate breast cancer microarray data. Left: one NKI all cohort; Middle: on NKI ER-negative cohort; Right: on GSE1456 all cohort

We further tested if this unique group of 201 SMGs (Additional file 1: Table S1) or its subsets is associated with patient outcome (survival time to be specific in this paper) using multiple publicly available breast cancer gene expression data. The results are shown in Fig. 7. The subsets were selected based on the IPA pathway annotation. Our test on NKI data suggested that the 201 SMGs are able to separate patients (based on K-means algorithm with *K* = 2) into two groups with significant survival time difference but cannot effectively separate the ER-negative patients. The 201 SMGs can be clustered into several functional/pathway groups based upon gene enrichment analysis using Ingenuity Pathway Analysis (IPA®). Among these groups, we found that the group of 27 genes with embryonic development functions performed the best, which can separate the ER-negative breast cancer patients into two groups with significantly different survival times (Fig. 6 Middle). In addition, this 27-gene set can also separate patients in the other dataset (GSE1456) as shown in Fig. 7 Right. Given the high enrichment of ER-negative patients in the Group 1, these results suggest that the 27 genes may form the core of the Group 1 SMGs. As a comparison, the SMGs unique to Group 2 were not able to separate the ER-negative patients with significantly different survival outcomes, and it does not performs as good as Group 1 SMGs on general population survival test (data not shown).

## Discussion

With recently rapid development in next-generation sequencing technology and computing capacity, huge amount of data in different modalities for cancer specimens have been accumulated in an amazing speed in public databases. Therefore, integrating and mining these data becomes a major challenge in the bioinformatics field currently. In this work, we developed a novel approach to integrate genomic, transcriptomic and clinical data of cancer patients, specifically to compare somatic mutations of patients based on their functional relationships in the context of gene expression profiles, thus tackling the challenge of low overlapping of mutated genes among cancer patients. By introducing the distance correlation metric to directly measure the relationship between two sets of genes affected by somatic mutations, we not only can cluster the patients into different groups with different clinical subtypes, but also visualize the clusters and identify group specific mutations. The power of using distance correlation freed us from comparing only gene pairs, but directly comparing gene list to list. The distance correlation captures not only linear relationship of the two lists as Pearson correlation does, but also reveals non-linear relationship as well, which covers the biological interaction in far more and deeper extent.

**Fig. 8:**
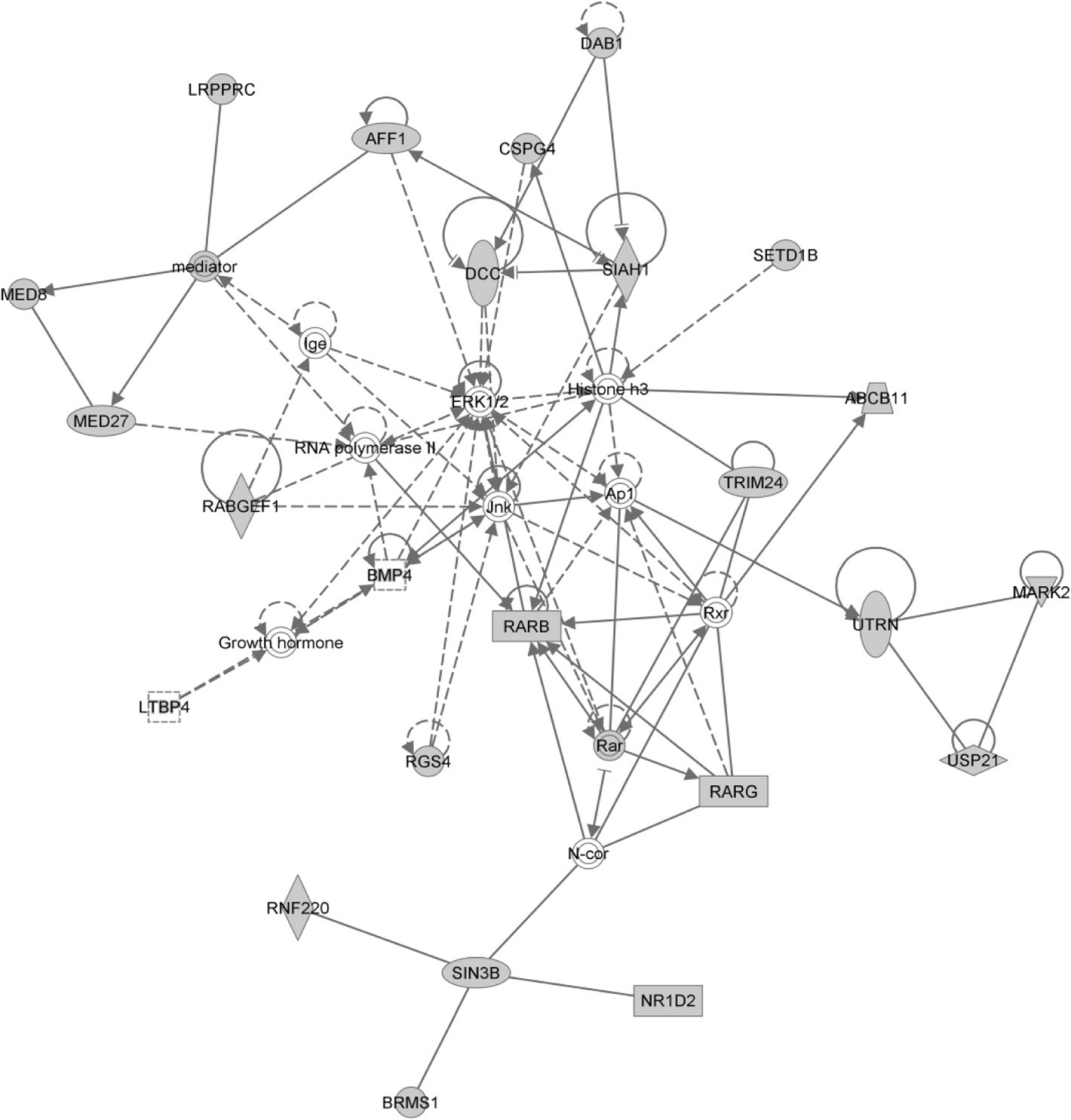
Group 1 genes enriched with embryonic development, organ development and morphology function (IPA)

Applying this approach on TCGA breast cancer patients reveals a group of patients who are mostly negative with one or more of the three breast cancer biomarkers (ER, PR, HER2) [12], and one third of the group are triple-negative subtype. Triple-negative breast cancer (TNBC) composes of 12–20 % of breast cancer patients [13]. It progresses more aggressively and does not respond well to hormone therapy. The rapid and aggressive progress of the disease course makes the prognosis of TNBC very poor [14] and the prediction difficult. After examining the group of patients we identified here, they harbor SMGs tightly interlinked each other and enriched with early stage cancer development. Among them, the 27 embryonic development genes form tight interaction networks as shown in the Fig. 8, and those genes can be used for breast cancer survival prognosis, especially for the poorly understood ER-negative cohort. TCGA database has not been curated long enough for this subtype of patients, therefore we did not test our findings on TCGA data. Instead, we chose two older GEO breast cancer microarray datasets. Unfortunately, the GEO datasets we tested does not contain enough TNBC patients, so we only tested on ER-negative cohort. The clustering results indicated that a portion of the triple negative patients maybe fundamentally different from the rest of the breast cancer patients due to the somatic mutations they harbor. Many of their genes shared common upstream regulators such as the drug for acute myeloid leukemia or estrogen, suggesting this group of people may benefit from other type of treatments that have not been administrated to TNBC patients. We suggested that the common upstream regulators and drugs interacting with these genes can provide insight on the development and treatment of TNBC patients. In addition, while among the 27 genes some of them are known to be associated with other cancers such as AFF1 [15], BMP4 [16], and TRIM24 [17], others such as MED27 is not widely know to be associated with cancers. Thus our work also generated new hypothesis on cancer related genes.

## Conclusions

In summary, a common challenge in studying complex diseases such as cancers is the lack of common genetic mutations among the patients. Besides pursuing commonly affected pathways, we provide a complementary approach for integrating the genotype data with transcriptome data to study the relationships between the genetic mutations at the functional level. While our main goal is on exploring the functional relationships of mutated gene groups, the identified genes may also serve as potential biomarkers for different subtypes of cancers. Currently due to the limitation of the data, we focus on the protein coding genes from the WES experiments.

In the near future, we plan to apply the same workflow to other cancer datasets in TCGA to further test the effectiveness of this method as well as identifying diseases in which such functional relationship can lead to meaningful stratification of the patients. With the cost of whole genome sequencing decreasing dramatically, it is expected that more somatic mutations on the non-coding regions and regulatory regions can be made available and the approach need to be expanded to accommodate such mutations.

## Abbreviations

BRCA, breast cancer; ER, estrogen receptor; GEO, gene expression omnibus; HER2, human epidermal growth factor receptor 2; IPA, ingenuity pathway analysis; MDS, multi-dimensional scaling; NCBI, National Center for Biotechnology Information; NGS, next generation sequencing; PR, progesterone receptor; RNA-seq, ribonucleic acid sequencing; SMG, significant mutant gene; TCGA, the cancer genome atlas; TNBC, triple-negative breast cancer; WES, whole genome exome sequencing

## Additional file

Additional file 1: Supplementary tables. This file contain two tables, the first table contain the SMGs in group 1 patients and their mutation frequencies among group 1 patients. The second table contain the patient IDs and their corresponding SMGs from TCGA BRCA. (DOCX 117 kb)
