# Supplementary material for: Integrative analysis of somatic mutations and transcriptomic data to functionally stratify breast cancer patients
Source: BMC Genomics. 2016 Aug 22;17(Suppl 7):513. doi: 10.1186/s12864-016-2902-0 (PMC5001235; doi:10.1186/s12864-016-2902-0)
Supplement: Additional file 1: — Supplementary tables. This file contain two tables, the first table contain the SMGs in group 1 patients and their mutation frequencies among group 1 patients. The second table contain the patient IDs and their corresponding SMGs from TCGA BRCA. (DOCX 117 kb) [file 12864_2016_2902_MOESM1_ESM.docx]

**Supplementary material for “Integrative analysis of somatic mutations and transcriptomic data to functionally stratify breast cancer patients”**

**Supplementary Table S1**

| **SMG in group I patients** | **Mutation frequency in group I patients** | **Unique to Group 1** |
| --- | --- | --- |
| TP53 | 40 | NO |
| SPEN | 2 | NO |
| PKD1L2 | 2 | YES |
| ZNF831 | 1 | NO |
| ZNF781 | 1 | YES |
| ZNF626 | 1 | YES |
| ZNF626 | 1 | YES |
| ZNF615 | 1 | YES |
| ZNF430 | 1 | YES |
| ZIM2 | 1 | YES |
| ZGLP1 | 1 | YES |
| ZFP3 | 1 | YES |
| ZDHHC8 | 1 | YES |
| ZDBF2 | 1 | NO |
| ZBTB40 | 1 | YES |
| ZBTB33 | 1 | YES |
| WWP2 | 1 | YES |
| WWC3 | 1 | NO |
| WDR64 | 1 | YES |
| WBSCR17 | 1 | YES |
| VPS11 | 1 | YES |
| VCL | 1 | YES |
| UTRN | 1 | YES |
| USP21 | 1 | YES |
| UBQLN1 | 1 | YES |
| UBE3C | 1 | YES |
| TRPC6 | 1 | YES |
| TRIM55 | 1 | YES |
| TRIM24 | 1 | YES |
| TRAPPC10 | 1 | YES |
| TRAPPC10 | 1 | YES |
| TMCO7 | 1 | YES |
| THNSL1 | 1 | YES |
| TGFBR2 | 1 | YES |
| TCEB3CL | 1 | YES |
| TBKBP1 | 1 | YES |
| TAF1L | 1 | NO |
| SYNE1 | 1 | NO |
| SYCP2L | 1 | YES |
| SVIL | 1 | YES |
| SVEP1 | 1 | YES |
| STARD13 | 1 | YES |
| SSPO | 1 | NO |
| SRBD1 | 1 | YES |
| SNRPN | 1 | YES |
| SLC25A35 | 1 | YES |
| SLC25A13 | 1 | YES |
| SLC22A3 | 1 | YES |
| SIN3B | 1 | YES |
| SILV | 1 | YES |
| SIAH1 | 1 | YES |
| SGK1 | 1 | YES |
| SETD1B | 1 | YES |
| SECTM1 | 1 | YES |
| SEC14L5 | 1 | YES |
| SAPS1 | 1 | YES |
| SAMD3 | 1 | YES |
| RTN4IP1 | 1 | YES |
| RNF220 | 1 | YES |
| RGS7 | 1 | YES |
| RGS4 | 1 | YES |
| RGS17 | 1 | YES |
| RGPD3 | 1 | YES |
| RFXANK | 1 | YES |
| REG1B | 1 | YES |
| RBM34 | 1 | YES |
| RBM12B | 1 | YES |
| RB1CC1 | 1 | YES |
| RB1 | 1 | NO |
| RARRES1 | 1 | YES |
| RARG | 1 | YES |
| RARB | 1 | YES |
| RABGEF1 | 1 | YES |
| PRKG2 | 1 | NO |
| PRKD1 | 1 | YES |
| PRDM8 | 1 | YES |
| PRDM14 | 1 | YES |
| PLA2G4E | 1 | YES |
| PGBD5 | 1 | YES |
| OR52M1 | 1 | YES |
| OR52H1 | 1 | YES |
| OR2B11 | 1 | YES |
| NSUN5C | 1 | YES |
| NRP2 | 1 | YES |
| NR1D2 | 1 | YES |
| NPDC1 | 1 | YES |
| NMT2 | 1 | YES |
| NMT1 | 1 | YES |
| NIF3L1 | 1 | YES |
| NFAT5 | 1 | YES |
| NDUFAF1 | 1 | YES |
| NCCRP1 | 1 | YES |
| MYO1G | 1 | YES |
| MYO10 | 1 | NO |
| MYH14 | 1 | NO |
| MUC6 | 1 | NO |
| MNDA | 1 | YES |
| MLL3 | 1 | NO |
| MIB1 | 1 | YES |
| MGMT | 1 | YES |
| MGC26647 | 1 | YES |
| MGAT5B | 1 | YES |
| METAP2 | 1 | YES |
| MED8 | 1 | YES |
| MED27 | 1 | YES |
| MARK2 | 1 | YES |
| MANEA | 1 | YES |
| MAGEC1 | 1 | YES |
| LYST | 1 | YES |
| LTBP4 | 1 | YES |
| LRRK1 | 1 | YES |
| LRRC41 | 1 | YES |
| LRPPRC | 1 | YES |
| LOC441806 | 1 | YES |
| LOC284395 | 1 | YES |
| LOC134505 | 1 | YES |
| LOC100289403 | 1 | YES |
| LOC100287962 | 1 | YES |
| LOC100287658 | 1 | YES |
| LOC100287244 | 1 | NO |
| LOC100130814 | 1 | YES |
| LCA5L | 1 | YES |
| KLRC1 | 1 | NO |
| KIAA1755 | 1 | YES |
| KIAA1267 | 1 | NO |
| KIAA1217 | 1 | YES |
| KCNK4 | 1 | YES |
| JAKMIP2 | 1 | YES |
| ITIH5L | 1 | YES |
| ITIH4 | 1 | YES |
| ISLR2 | 1 | YES |
| INHBC | 1 | YES |
| IL11RA | 1 | YES |
| IGSF5 | 1 | NO |
| IGFL4 | 1 | YES |
| HOOK2 | 1 | YES |
| HERC4 | 1 | NO |
| HEATR3 | 1 | YES |
| GRIA3 | 1 | NO |
| GOLGA3 | 1 | YES |
| GNPTAB | 1 | NO |
| GNAT2 | 1 | YES |
| GJA10 | 1 | YES |
| GALNT13 | 1 | YES |
| FXR1 | 1 | YES |
| FLG2 | 1 | YES |
| FGFBP1 | 1 | YES |
| FAM54B | 1 | YES |
| FAM53C | 1 | YES |
| FAM53B | 1 | YES |
| FAM117B | 1 | YES |
| EPHA3 | 1 | YES |
| ENSG00000237362 | 1 | NO |
| ENSG00000225607 | 1 | YES |
| EMR2 | 1 | YES |
| EML4 | 1 | YES |
| ELTD1 | 1 | YES |
| DSEL | 1 | YES |
| DOPEY2 | 1 | YES |
| DOCK6 | 1 | YES |
| DNAJC6 | 1 | YES |
| DNAJC1 | 1 | YES |
| DNAH9 | 1 | YES |
| DNAH7 | 1 | YES |
| DNAH10 | 1 | YES |
| DMKN | 1 | YES |
| DKFZp761E198 | 1 | YES |
| DCHS2 | 1 | YES |
| DCDC5 | 1 | YES |
| DCC | 1 | YES |
| DAO | 1 | YES |
| DAB1 | 1 | YES |
| CYP7B1 | 1 | YES |
| CYP2D6 | 1 | YES |
| CXorf30 | 1 | YES |
| CSPG4 | 1 | YES |
| CRYGC | 1 | NO |
| CREBZF | 1 | YES |
| COX10 | 1 | YES |
| COL25A1 | 1 | YES |
| CNOT1 | 1 | YES |
| CDKL2 | 1 | YES |
| CD97 | 1 | YES |
| CD248 | 1 | YES |
| CCR7 | 1 | YES |
| CCDC57 | 1 | YES |
| CCDC39 | 1 | YES |
| CAMK2D | 1 | YES |
| C9orf93 | 1 | YES |
| C9orf144B | 1 | YES |
| C6orf170 | 1 | YES |
| C2orf51 | 1 | YES |
| C1orf192 | 1 | YES |
| C17orf71 | 1 | YES |
| C17orf104 | 1 | YES |
| C15orf2 | 1 | YES |
| C12orf63 | 1 | YES |
| C10orf47 | 1 | YES |
| BRWD1 | 1 | NO |
| BRMS1 | 1 | YES |
| BMP4 | 1 | YES |
| BCL9 | 1 | NO |
| BCAS1 | 1 | YES |
| BAZ2A | 1 | NO |
| BAG3 | 1 | YES |
| ATP2B2 | 1 | YES |
| ATP1B3 | 1 | YES |
| ATP10B | 1 | YES |
| ATAD5 | 1 | YES |
| APOBEC2 | 1 | YES |
| ANO9 | 1 | YES |
| ANKRD50 | 1 | YES |
| ANKRD13C | 1 | YES |
| ANKK1 | 1 | YES |
| AKR1C2 | 1 | YES |
| AGBL5 | 1 | YES |
| AGBL1 | 1 | YES |
| AFF1 | 1 | YES |
| ADCY10 | 1 | YES |
| ADCK1 | 1 | YES |
| ADAMTSL3 | 1 | YES |
| ACVR1B | 1 | YES |
| ACTL6B | 1 | YES |
| ACSF2 | 1 | YES |
| ACAP2 | 1 | YES |
| ABCF2 | 1 | YES |
| ABCB11 | 1 | YES |
| AASS | 1 | YES |

**Supplementary Table S2 (patients from Group 1 are highlighted)**

| **Patient ID** | **SMGs** |
| --- | --- |
| TCGA-A1-A0SD-01 | TLR5, PNLIPRP2 |
| TCGA-A1-A0SE-01 | RBM26, MED23 |
| TCGA-A1-A0SH-01 | ATPIF1, PLCE1, TAS2R46, AHCTF1, BRCA1, CSMD1, ESCO1, FBXO4, H2BFWT, HYDIN, RHCG |
| TCGA-A1-A0SJ-01 | CHML, SPEN, TNRC6A, ENSG00000240720, OFD1 |
| TCGA-A1-A0SK-01 | GTF3C1, OBFC2B, SLITRK4, UGT2B15 |
| TCGA-A1-A0SM-01 | AHDC1, EGR3, SEZ6L2, SMARCA1, KCNH7, CDY2A, DCST1, TBC1D8 |
| TCGA-A1-A0SO-01 | ALKBH8, C1orf58, CHRM5, FRY, GRIA1, KIAA1751, MACF1, NBEAL2, PHF2, PLEK2, RDH5, SFPQ, AMZ1, GOLGB1, KIAA1107, PMEPA1, SSC5D, TNFRSF11B, ZDHHC5 |
| TCGA-A2-A04N-01 | CD55, MAP3K1, ASXL1, RAD9A |
| TCGA-A2-A04P-01 | HLA-A, HTR1A, ILKAP, OR7A10, TP53, CDKL1, HEYL, SEMA4D, SOS1 |
| TCGA-A2-A04Q-01 | MST1, GPI, DNAH14, NF1 |
| TCGA-A2-A04R-01 | COL18A1, LCE2A, CELSR1, GPR179, HNRNPA3, TM7SF3 |
| TCGA-A2-A04U-01 | ATP2B2, TP53 |
| TCGA-A2-A04V-01 | KIAA1267, MIF4GD, PLEKHA2, RNF212, TMIGD2 |
| TCGA-A2-A04W-01 | TMC5, CCT8L1, CHD4, CRYGC, GNPTAB, IGLON5, MMS19, NKD2, NLRP13, SEMA3C, SLC34A3, ZNF667 |
| TCGA-A2-A04X-01 | UBE2Q1, SARM1 |
| TCGA-A2-A04Y-01 | ARNTL, EGR3, KIAA0232, CEP164, PXDN, PCSK5 |
| TCGA-A2-A0CL-01 | C20orf72, CARD10, CEP135, MRC2, REV1 |
| TCGA-A2-A0CM-01 | TP53, PRDM14, ATP1B3, BMP4, CCDC39, CDKL2, MGMT |
| TCGA-A2-A0CP-01 | BAHCC1, C19orf40, C9orf140, CMIP, IQSEC1, LCMT2, LOC100289287, ACOXL, CCND3, EPB41L2, GAPVD1, LRGUK, MAP3K1 |
| TCGA-A2-A0CS-01 | EFR3A, LLGL2, MDGA1, CDKN1B, MLL3, BMPR2, RAPGEF3 |
| TCGA-A2-A0CT-01 | FRMPD3, EML3, C3orf34, INPP5F, STX10 |
| TCGA-A2-A0CU-01 | KIAA2022, MST1P9, NRBP1, IRF2BP2, GNB3 |
| TCGA-A2-A0CV-01 | GATA3, FAM179B |
| TCGA-A2-A0CW-01 | C9orf93, DMKN, ITIH5L, RTN4IP1, GNAT2, CNOT1, TP53 |
| TCGA-A2-A0CX-01 | PPHLN1, T, ARHGAP5, CABYR, CALCOCO1, DLG1, EWSR1, FAM196A, FASTKD3, GGT1, HSPA9, NEK10, NSUN3, PION, RABEP1, RGL3, TMEM89, ZFHX3 |
| TCGA-A2-A0CZ-01 | BAT2, SPRED2, GATA3, TMEM132B |
| TCGA-A2-A0D0-01 | PARP9, FAM166B, ARFGEF1, TNKS1BP1, TP53 |
| TCGA-A2-A0D1-01 | ECH1, GYLTL1B, SEZ6L, NARFL, UBC, GPR98, OR5A1, TP53, ZSCAN21 |
| TCGA-A2-A0D2-01 | ENSG00000225607, JAKMIP2, KCNK4, MAGEC1, SEC14L5, TP53, TRIM55 |
| TCGA-A2-A0D3-01 | DNAJC2, MLL3, ZFP30, GATA3, BAI3, C1orf129, NLRP3 |
| TCGA-A2-A0D4-01 | CHST2, PDS5A, KIAA0319, FAM90A1 |
| TCGA-A2-A0EM-01 | SGCZ, MAGEL2, MYEF2 |
| TCGA-A2-A0EN-01 | MLL3, PSMD1, TBX4 |
| TCGA-A2-A0EO-01 | ENPEP, RDBP, MR1 |
| TCGA-A2-A0EQ-01 | DOCK3, CHRNA10 |
| TCGA-A2-A0ER-01 | CEP164 |
| TCGA-A2-A0ES-01 | DNAH8, EEF1A1 |
| TCGA-A2-A0ET-01 | MYB, DHRS2, IQGAP2, IWS1, TMEM132B |
| TCGA-A2-A0EU-01 | FOXA1, MAP3K1 |
| TCGA-A2-A0EV-01 | NCOR1, TRAK1, ZNF420 |
| TCGA-A2-A0EX-01 | ESPL1, KIAA2013, LOC221710, TRIM11, WHSC1, CDH1, FXYD5, HK1 |
| TCGA-A2-A0EY-01 | C22orf30, CCDC14, STRN, UMODL1, ACTR6, CDC42BPA, CDC42BPB, COL4A5, DMXL2, IL6, LMAN1L, MUC17, PABPC3, SLC38A1, SMC6, ZNF587 |
| TCGA-A2-A0ST-01 | EFHD2, FIP1L1 |
| TCGA-A2-A0SU-01 | ACTG1, GIMAP7, IFT122, TMEM213, EFCAB4A, GATA3, AZI1, BRCA2 |
| TCGA-A2-A0SW-01 | MCTP2, MERTK, SERPINA4, MYEOV2, ZC3H6 |
| TCGA-A2-A0SX-01 | PPEF1, STAG2 |
| TCGA-A2-A0SY-01 | GPS2, MYB, CDH1, SFRS15, BAT2L2, DMRT1, FAM188A, HRC, NOX4, OR14I1, PRMT10 |
| TCGA-A2-A0T0-01 | BAI1, ENSG00000188014, EPN2, NUP98, PDE6G, PLCL2, WDR11, SET, LOC100129786, BRCA2, DNAH5, FCGR1B, HERC5, LRRC70, SLITRK6, SMARCA4, TTN, ZNF438 |
| TCGA-A2-A0T1-01 | FAM160A2, LRRC10, MAGED1, MAP3K9, NPTN, EML4 |
| TCGA-A2-A0T2-01 | ABHD6, LAMC1, QPCT, RBM47 |
| TCGA-A2-A0T3-01 | TP53, C7orf71, BAT2L2, COL6A3, LHFPL4 |
| TCGA-A2-A0T4-01 | AGAP7, CTPS2, CDH1, CCR2, ENSG00000232637, ZNF618 |
| TCGA-A2-A0T6-01 | PPP2R4, CDH1, MED23 |
| TCGA-A2-A0T7-01 | RBM15, CCDC146, C14orf145, ANKRD2, ATM |
| TCGA-A2-A0YC-01 | ZNF90, NCOR1 |
| TCGA-A2-A0YD-01 | CATSPER2, CDH1, CYFIP2, DENND4C, GLCE, MINPP1, NAV2, NUP214, OR7A5, SAMD3, TAS2R30, UBA6, URB1, MAP3K1, PKD1 |
| TCGA-A2-A0YF-01 | C18orf55, ZNF486, ECHDC2, CTCF, RUNX1 |
| TCGA-A2-A0YG-01 | CYP2D7P1, BAZ1B, MBD6 |
| TCGA-A2-A0YH-01 | FLNA, PYGB, SCN9A, ZNF532, TPM2, ENSG00000188014, FAM75A2, FSHR, C6orf35 |
| TCGA-A2-A0YI-01 | SPEN |
| TCGA-A2-A0YJ-01 | ENSG00000237362, UBQLN1, ABCF2, TAF1L, TP53, ZNF430 |
| TCGA-A2-A0YK-01 | ARHGAP29, ARHGEF7, ARMC10, CCDC125, CDH1, COPS6, DYNC2LI1, MAP3K1, MID2, PTPRK, WARS2, YTHDF1, ZCCHC17, HAUS3, PUM1 |
| TCGA-A2-A0YL-01 | NF1, MAP2, MSX2 |
| TCGA-A2-A0YM-01 | SRBD1, TP53, ATAD5, NMT2, PRKG2, SVIL |
| TCGA-A7-A0CD-01 | PLXNA1, ZC3H12A |
| TCGA-A7-A0CE-01 | HERC1, LOC653720, SALL1, SLC27A3, CCDC146, ENSG00000176540, ANK1, FAM171A1, MYCT1, RTN4RL1, STRN4, TRERF1 |
| TCGA-A7-A0CG-01 | OR13A1, SEMA3B, MRPL43, CXorf58, ZCCHC16 |
| TCGA-A7-A0CH-01 | ABCA1, WNT7A, TBX3, STAG2 |
| TCGA-A7-A0CJ-01 | SLCO5A1, TRO, CCDC30, PCTK3, POLQ |
| TCGA-A7-A0D9-01 | LOC100288105, WDR13, LRRN3, MAPK8 |
| TCGA-A7-A0DA-01 | DNAH11, DPY19L2, LOC100132941, MRFAP1, NFKB2, SMC1B, SYNGAP1, TRAF7, ASB7, CEP350, FAM135B, LILRA6, SMTNL1, TSPAN12, VCX3B |
| TCGA-A7-A0DB-01 | MAP3K1, NOL6, ACAA1, ZKSCAN1 |
| TCGA-A7-A13D-01 | KIAA1217, MYO10, PRDM8, RGS17, CD97, EMR2, FLG2, RARG, RGS7, SIAH1, TP53, ZNF615 |
| TCGA-A7-A13F-01 | FAM46A, C21orf57 |
| TCGA-A8-A06N-01 | ARID1A, ZNF782 |
| TCGA-A8-A06O-01 | DNAH6, DYNC2H1, LARP4B, PTEN, ZNF292, BLM, PON1, STAT5A |
| TCGA-A8-A06P-01 | GOLGA6L3, MAP3K1, TCF19, SYNCRIP |
| TCGA-A8-A06Q-01 | C11orf45, TMEM63B, ZNF862, ZNF536, CCDC111, CD1E, CENPE, CHD2, CLEC4M, EBF1, FLG, INSL5, KDM4DL, MYCBP2, NIN, REP15, TMEM88B, TRPV2, ZMYM4 |
| TCGA-A8-A06T-01 | ASXL2, KRT39, GATA3, TJP1 |
| TCGA-A8-A06X-01 | CBLC, KRTAP5-5, PARD3B, RTTN, MLL3, FCHSD1, HCLS1, MGAT5, TAF3 |
| TCGA-A8-A06Y-01 | KDM6A, OR4C45, SCYL2, RPS21 |
| TCGA-A8-A06Z-01 | LOC729225, OR5H15, PLEKHH1, ZFP28 |
| TCGA-A8-A075-01 | ATRX, MACF1, ZNF501, ARHGEF12, DDX27, NRP1, SF3B1 |
| TCGA-A8-A076-01 | AOC3, DUSP16, TRAK2, ZNF716 |
| TCGA-A8-A079-01 | HECW1, RP2 |
| TCGA-A8-A07B-01 | GNPTAB, HOOK2, KIAA1267, TP53, NFAT5, IL11RA, SAPS1, STARD13, TRIM24 |
| TCGA-A8-A07C-01 | COL2A1, PARP10, RUFY3, BNC2, COL18A1, HERC4, HKR1, IFIT1L, ITPKB, MYO5C, ODZ1, RETSAT, TAS1R3 |
| TCGA-A8-A07E-01 | ANKRD28, CCR5, FMN2, GLE1, TLN1, ABCB6, FARP2, MYH7, CDKN1B, DYNC1H1, IFT122 |
| TCGA-A8-A07F-01 | CDH1, MAP3K1, HIST1H2AM, WTAP |
| TCGA-A8-A07G-01 | RXFP1, TAB2, CAPSL, ENG, HNRNPA2B1, KIAA1383, RAB40A, WSCD2, ZCCHC16 |
| TCGA-A8-A07J-01 | ARID1A, CCR10 |
| TCGA-A8-A07L-01 | ABL1, DNAJC11, GSTCD, TAB3, C12orf68, DYNC2H1, FSIP2, HIST1H2AI |
| TCGA-A8-A07O-01 | LOC55908, MLYCD |
| TCGA-A8-A07P-01 | ATM, RAD21, L3MBTL4, CMPK2, MLL, PPEF1, PSMD5 |
| TCGA-A8-A07R-01 | C10orf25, EBPL, GPS2, HOMER3, HSPG2, LRSAM1, MAP1A, SLC10A3, SLC35A2, DOCK4, CTNND2, DOCK2, TMEM223, ADPRH, CHD4, DENND5A, LIG4, MDN1, NRP1, POLA1, SLC13A1, TMED6, VPRBP, ZNF233 |
| TCGA-A8-A07U-01 | CCDC57, ISLR2, TP53, ANKK1 |
| TCGA-A8-A07W-01 | EXT1, PRF1, SPRY3 |
| TCGA-A8-A081-01 | ZNF510, KRT19, USP15, GTF2IRD2, OSBPL9 |
| TCGA-A8-A082-01 | MAP3K1, VIPAR, CPA4 |
| TCGA-A8-A085-01 | BPI, BRCA2, DDX11, PIWIL2, SNAI2, LOC100128816, LOC100288624, MYCBP2, MYH7, OR6N1, SGCA |
| TCGA-A8-A086-01 | LYSMD3, MAP3K1, SBF1, TARBP2 |
| TCGA-A8-A08B-01 | RAPGEF4, MED23, SH3PXD2A |
| TCGA-A8-A08F-01 | CGNL1, HNRNPF, HSF2, NF1, PLA1A, TRIM46, B4GALT3, ASXL2, FBXW4, NGEF, PCDHA8 |
| TCGA-A8-A08H-01 | KIF26B, MEFV, SRPX2, ZBTB48, ADCY9, SLC12A4, CD5L |
| TCGA-A8-A08I-01 | ALOXE3, BIN1, C14orf25, MYO9B, SLC25A39 |
| TCGA-A8-A08J-01 | GATA3, GTF2IRD2B |
| TCGA-A8-A08L-01 | C12orf4, GRIN2A, KCNK2, MTBP, AFF2, CRAT, ELF1, MTR, ADAMTS9, ATP6V1G2, C12orf50, C6orf174, CEACAM21, CYP2C18, ITPR3, JAK2, MUC17, RIPK3, SERPINA1, TLN1, 1-Dec |
| TCGA-A8-A08O-01 | HSP90AB1 |
| TCGA-A8-A08P-01 | BAZ2A, TP53, ACAP2, MLL3, ZNF831 |
| TCGA-A8-A08R-01 | BAG3, HERC4, KLRC1, LRPPRC, SSPO, NSUN5C, TCEB3CL, TP53, ADCY10, ANKRD13C, ANKRD50, BRWD1, FAM54B, GALNT13, OR52M1, RNF220, SYNE1, WDR64 |
| TCGA-A8-A08T-01 | PRPF4, C1orf222, AHNAK2, APOL1, PPARGC1B, TOX4 |
| TCGA-A8-A08X-01 | TMEM110, FNDC3A |
| TCGA-A8-A090-01 | FLNB, LAMA2, MED7 |
| TCGA-A8-A091-01 | NCOR2 |
| TCGA-A8-A092-01 | CDKN1B, PAX2, CCNB1, UBE2J1, CYBB, GLE1, OR51A4, SORBS1, UQCR10, WDR82 |
| TCGA-A8-A093-01 | BAIAP2, AKAP11, DGUOK, IKZF4, LAMP2, LOC100287841, NCOR1, OSCP1, PPP1R9A, PRMT10, PTPN13, SMARCA5, ZNF180, NFASC |
| TCGA-A8-A094-01 | ACSS3, ASPM, FTSJD1, LEMD3, MLL3, OBSCN, SMAD4, SMC6, TP53, ATF6, BSN, C1orf85, C2orf68, DSP, RC3H2, ZBTB6, ZNF350, ZNF45, TMEM87B |
| TCGA-A8-A095-01 | ENSG00000226997, ASPA, CTH, RB1, RPH3AL, SEC24B, SFMBT1, SYNE1, TRNAU1AP |
| TCGA-A8-A097-01 | CUX1, DPAGT1, HIST1H3C, PUF60, TP53, MLL4, SLC16A2, MICAL2, PECI, PHLPP1 |
| TCGA-A8-A09A-01 | KLF17, SEMA7A, ADAM19, BTRC, CCDC112, EPB41L1, EXT1, HIPK2, JAK2, USP34 |
| TCGA-A8-A09B-01 | ESPL1, TNRC6B, COL5A3, MAP3K1 |
| TCGA-A8-A09C-01 | SORBS3, DBH, VWA3A, ABCA13 |
| TCGA-A8-A09D-01 | GRLF1, RFC4, TSPAN33, RPS6KL1 |
| TCGA-A8-A09G-01 | AADACL2, C1orf89, EIF4A2, OR2L3, RNF213, WDFY3, C10orf18, DISP2, DUSP16, EFCAB7, FLRT3, LYPD6, OR5D13, PCDHGA9, RNASE9, RRP15, RYR2, SPTBN2, TMEM38A, TMEM48, TNPO1, ZNF573, C9orf119, MCM6 |
| TCGA-A8-A09I-01 | WDR33, SPEN, CA11, MAGEA12, NCOR1, SCML1, SMYD3 |
| TCGA-A8-A09N-01 | PRDX1, TYW1B |
| TCGA-A8-A09Q-01 | CTH, ENSG00000197041, GRIK2, MSH4, SBF2, SDR16C5 |
| TCGA-A8-A09R-01 | SYNE2, GATA3, TBX3 |
| TCGA-A8-A09V-01 | SNAP23, SPACA1 |
| TCGA-A8-A09X-01 | CDH1, C5orf20 |
| TCGA-A8-A0A1-01 | AKAP12, CASC5, SOX10, ATP6V0A2, COL24A1 |
| TCGA-A8-A0A2-01 | NCOR1, PCDHB5 |
| TCGA-A8-A0A4-01 | FAM129A, PLXDC1, ADCY4, CDH1, PXDN, TBX3, LPAR4, TLN2 |
| TCGA-A8-A0A7-01 | CCDC11, CTTNBP2, EP300, HAT1, OR8D4 |
| TCGA-A8-A0A9-01 | AKD1, CDH1, FBXO18 |
| TCGA-A8-A0AB-01 | AGAP7, CLSPN, TRIP6, CDH1 |
| TCGA-A8-A0AD-01 | PCDHGA9, ZFHX3, NF1, IFT52 |
| TCGA-AN-A041-01 | DALRD3, GATA3, TFDP2 |
| TCGA-AN-A049-01 | DNPEP, FBXO21, MAP3K1, KLHL15 |
| TCGA-AN-A04A-01 | GATA3, C2orf89, LOC100286978, MKL1 |
| TCGA-AN-A04C-01 | PEG10, UGGT2, PCDH9 |
| TCGA-AN-A04D-01 | RNF43, SNTG1, ATAD2B, OR10S1, SF4, ZNF841 |
| TCGA-AN-A0AL-01 | ABI3BP, ACY1, MX2, MYPN, NF1, ORM1, PCDHAC1, USPL1, C12orf35, HRNR, KCNT2, NRXN3, PCDHGB4 |
| TCGA-AN-A0AM-01 | ZNF724P, GATA3, PPIB, FBXL18, MYO1A, RBBP5 |
| TCGA-AN-A0AT-01 | INSRR, PREPL, TNFRSF10D, ZFYVE1, CREBBP, CTCF, TBRG1 |
| TCGA-AN-A0FD-01 | CEP290, WAC, GATA3, DOCK11, MAP4K1, RWDD3 |
| TCGA-AN-A0FF-01 | GNAS, C1orf187, NR1I3 |
| TCGA-AN-A0FJ-01 | AGBL5, ATP10B, NIF3L1, TP53, DNAJC6, HEATR3, ZGLP1, ZNF626 |
| TCGA-AN-A0FK-01 | PAX8, TTLL12 |
| TCGA-AN-A0FL-01 | BRWD1, CCBL1, HEATR7A, KIAA1797, MARVELD3, MTMR15, SH3BP4, SPTBN5, AFF2, ASPA, BCL2L12, C16orf62, C8orf31, DFNA5, FAM49A, GNAT3, OSBPL3, RANBP3L, ZBTB24, ZDBF2 |
| TCGA-AN-A0FN-01 | LOC100289444, MLL3, TNXB, PPP1R3F, LRRN4 |
| TCGA-AN-A0FS-01 | FAM71E2, FGFR4, IRS1, LOC400986, LRRC46, TBC1D10C, LPGAT1, TBX3, MAP1A, ODZ2, SVEP1 |
| TCGA-AN-A0FT-01 | ELOVL5, C9orf91, NFASC, SHROOM2, EXOC6, COLEC12, DMD, KCNH8, KCNN2, MOS, NEDD9, PLEKHM3, ZFP106 |
| TCGA-AN-A0FV-01 | CCDC88A, MED13L, SERPINC1, TNPO1, GNRH2, SLC15A4, C2orf44, DHX30, RFX7 |
| TCGA-AN-A0FW-01 | CCPG1, TMEM82, ATP2A1, EMILIN3, FAM168A, HCN1, NFIC, STAT5A, SYCP2, HOOK3 |
| TCGA-AN-A0FX-01 | AP2M1, ARID1B, CAMKK1, FLNC, ITPR1, KIAA0947, KLHDC1, KRT17, TMEM201, WNT11, TLE6, ACTL7B, C4orf52, DGKE, LOC100130982, LRDD, MBLAC1, MYOF, SF3B2, SOX14, ZNF296, CD79A, FAM135A, INPPL1 |
| TCGA-AN-A0FY-01 | PER3, PLD4, PTPRCAP, PLCG1, RB1 |
| TCGA-AN-A0FZ-01 | PTPRD, SGPL1 |
| TCGA-AN-A0G0-01 | PKD1L2, ACVR1B, SNRPN, TGFBR2, TP53 |
| TCGA-AN-A0XL-01 | ZNF292, ANPEP, CHL1, IFNE, INHBA, KIAA1012 |
| TCGA-AN-A0XN-01 | ASXL3, EFEMP1, LIN9, LRCH3, USP9X, ACSM2B, C10orf68, PLOD2, TAF1, ZSCAN23 |
| TCGA-AN-A0XO-01 | IL1R2, TAP1, HSD17B13, AMPD1, NAV3 |
| TCGA-AN-A0XP-01 | ELL2, HSD17B4, MLL3, CD2, FAT1 |
| TCGA-AN-A0XR-01 | GATA3, MAP2K4, TBX3, TRIM77 |
| TCGA-AN-A0XS-01 | MAP3K1, TAF3, THNSL2, XRCC6BP1, ATP9A |
| TCGA-AN-A0XU-01 | ANO9, CREBZF, DOCK6, ITIH4, MYH14, MYO1G, SETD1B, SPEN, TP53, TRPC6, ZNF626, UBE3C, MGC26647, C17orf71, DOPEY2, SECTM1 |
| TCGA-AN-A0XV-01 | INADL, NCAM2, PSMD1, CEP120, MLL3, OR2B3, LARP1B |
| TCGA-AN-A0XW-01 | ATIC, CABIN1, CACNA1G, DMXL1, EZH2, FAM116A, FAM86C, FRY, GMFB, IL23R, KIF5B, MUC17, PCOLCE2, PKN3, PRMT2, RC3H2, RDH11, SERPINA11, SIK3, TMEM132D, TNFSF4, TOP3A, ZNF700 |
| TCGA-AO-A03L-01 | CRHR1, KCNA7, PRRX1 |
| TCGA-AO-A03N-01 | KLHL17, MED12, AR, C17orf78, DLC1, GGCT, KRT38, L3MBTL3, MAP3K4, OPHN1, OBSCN |
| TCGA-AO-A03O-01 | RUNX1, SFI1, ACACA, GPR26, IARS, TFCP2 |
| TCGA-AO-A03R-01 | PCDHGA9 |
| TCGA-AO-A03T-01 | ARMCX2, DST, ENSG00000181359, JTB, PCDH19, TTN, MAP2K4, DHX15, VPS37A, LPCAT4 |
| TCGA-AO-A03U-01 | AP3D1, GPR98 |
| TCGA-AO-A03V-01 | C9, HEATR1, MAP3K11 |
| TCGA-AO-A0J2-01 | GTSE1, HAUS5, IL28RA, UPB1, HERC2, FAR1, ABCB11, WDR35 |
| TCGA-AO-A0J3-01 | CELSR3, FAM20C, GATA3, MAGEL2, RNF111, ADAMTS17, C6orf118, GRIN2B, NID2, TTN |
| TCGA-AO-A0J4-01 | LCN10, SLIT2, C20orf123, FAM190A, RGS22 |
| TCGA-AO-A0J6-01 | BCL9, C12orf63, DNAH10, DSEL, FGFBP1, GRIA3, IGFL4, TP53, WWC3, CYP2D6, FAM53B, LTBP4, NR1D2 |
| TCGA-AO-A0J7-01 | PGR, ALOX5, ENSG00000231452, TRMT2B |
| TCGA-AO-A0J8-01 | AMMECR1, CDH1, SRD5A2, MBD6, TSC22D1 |
| TCGA-AO-A0J9-01 | CDH1, EDEM3, OBSL1, GLOD4, MACF1, ZNF259 |
| TCGA-AO-A0JA-01 | GPS2, LYSMD3, NEU2, RLF, CSMD1, PTEN |
| TCGA-AO-A0JC-01 | ABCA1, TRBV30 |
| TCGA-AO-A0JD-01 | EYS, BEST2, FAT3, KIAA0195, KIAA0652, MERTK, OR2L2, PCM1, SEMA5A, SPHKAP, ZFR |
| TCGA-AO-A0JE-01 | DIP2C, KIAA0430, IRF9, MLL3, OR4D10, ZNF620 |
| TCGA-AO-A0JF-01 | MAP3K1, MLL3, CCDC146, MAGED2 |
| TCGA-AO-A0JG-01 | DSCAML1, FLII, KLC4, MAP3K13, GATA3 |
| TCGA-AO-A0JI-01 | PCNXL2, OVCH1 |
| TCGA-AO-A0JJ-01 | CDH1, MED23, ZNF655, UNC13C |
| TCGA-AO-A0JL-01 | FXR1, PGBD5, PLA2G4E, ZDHHC8, INHBC, TP53 |
| TCGA-AO-A0JM-01 | PHLPP2, CHST9, ATM, C7orf60, GOSR1 |
| TCGA-AO-A124-01 | LYST, MNDA, DAB1, RARRES1, ADAMTSL3, CCR7, CRYGC, LRRK1, TP53 |
| TCGA-AO-A125-01 | CDH10, JAK2, LOC643677, HYDIN, MLL3, PRPSAP2 |
| TCGA-AO-A129-01 | ACTN4, ATM, GPR97, PYGM, IQGAP2, REM1, TP53I3 |
| TCGA-AO-A12A-01 | GATA3, CWF19L2, ERBB3 |
| TCGA-AO-A12B-01 | CTCF, PTPN14, TRHDE |
| TCGA-AO-A12D-01 | TP53, OR2B11 |
| TCGA-AO-A12E-01 | XPO4, ZNF438 |
| TCGA-AO-A12F-01 | LOC644950, IGSF5, SESN2 |
| TCGA-AO-A12G-01 | C10orf119, CCAR1, PTEN, GLI3, MTOR |
| TCGA-AO-A12H-01 | USP9X, KDM4D, OBSCN |
| TCGA-AQ-A04H-01 | GLYR1, TYRP1, GATA3, ATP11A, COL19A1, NIN, OTX2 |
| TCGA-AQ-A04J-01 | FOXP4, MAGEA10, MAP3K12, CLIP2, DNAH17, GPR153, MLC1, ISOC1, SOBP |
| TCGA-AQ-A04L-01 | C19orf39, ENTPD7, HTR2B, PHF8, RPAP2, ZBTB49, RNLS, SLC5A9, UBR5, ZNF720 |
| TCGA-AR-A0TP-01 | ATP2C1, IQCF6, LMTK3, LPHN3, PLA2G6, SEMA3D, ABCC9 |
| TCGA-AR-A0TQ-01 | GATA3 |
| TCGA-AR-A0TR-01 | APLP2, NELL2, METTL2A |
| TCGA-AR-A0TS-01 | DCHS2, USP21, RBM12B, TP53 |
| TCGA-AR-A0TT-01 | KCNB2, NBEA, SLC35F2, ZSCAN12 |
| TCGA-AR-A0TU-01 | C14orf37, HDAC2, HOXA4, ODF1, SCN1A, MET, ZNF300, ADD2, NF2 |
| TCGA-AR-A0TV-01 | SLC4A5, DEAF1, NANS, PCDH1, TDRD10 |
| TCGA-AR-A0TW-01 | GATA3, CDC42BPA, KIAA1543, MTMR14, NCOR1, PCDHA13 |
| TCGA-AR-A0TX-01 | C11orf49, EPS8, ARID2, ATP13A4, C14orf145, C6orf97, CCDC47, COL5A3, CYP4Z1, DLG1, FAM13C, GPR83, LOC100271715, MAP3K4, MLL, MUC16, PHC3, POLR3E, RANBP2, RIC8A, ROR1, RTN3, SETD2, SPEN, STK17B, TET2, VCP, WDR7, ZCWPW1, SOX9 |
| TCGA-AR-A0TY-01 | FRRS1, LAMA3, LOC100287993, LRRC10, MUC5B, NR5A2, TRMT2A, USP20, CSRP2BP, GATA3, LRG1, PKHD1, CPNE4, EEA1, RELN |
| TCGA-AR-A0TZ-01 | CST2, OC90, PCSK2, TRAPPC9 |
| TCGA-AR-A0U0-01 | VWA3A, ARID2, LOC647166, PKD2L1, SELL |
| TCGA-AR-A0U1-01 | AP2M1, BMPR2, PARP8, SSPO, SYT17 |
| TCGA-AR-A0U2-01 | FYCO1, GATA3, DOPEY1 |
| TCGA-AR-A0U3-01 | C15orf2, PKD1L2, RGPD3, TP53, C17orf104, DAO, NMT1, RFXANK |
| TCGA-AR-A0U4-01 | BCL11B, IL6, KCNS2, MOCOS, TAAR1, BRCA1, AK7, C4BPB, CAST, DNAI2, ENSG00000187900, FAM177B, LOC729898, ZNF420, ZNF519 |
| TCGA-AR-A1AH-01 | FAM193B, KIAA0182, TP53, PADI2, KHSRP, MYH14, TLL1, TM6SF2 |
| TCGA-AR-A1AL-01 | CDH1, MYBPHL |
| TCGA-AR-A1AN-01 | TP53, DNAJC1 |
| TCGA-AR-A1AQ-01 | DKFZp761E198, LRRC41, TP53, WWP2, APOBEC2, ZBTB33, C1orf192, DNAH7, RB1 |
| TCGA-AR-A1AR-01 | LOC643677, RRAGB, CTNNA3, BAZ2B, KLRF1, LOC390424, TINAG, UCK2 |
| TCGA-AR-A1AV-01 | EIF3I, GATA3, IL12A, MAGI2, DNAJB5, PRKG2, VCAN |
| TCGA-AR-A1AW-01 | BCMO1, MPZL1 |
| TCGA-AR-A1AY-01 | AFF1, C9orf144B, SLC25A35, TP53, C6orf170, CXorf30, LOC100287658, REG1B, VCL |
| TCGA-B6-A0I2-01 | TP53, ADCK1, AGBL1, DCC, MARK2, MIB1, PRKD1 |
| TCGA-B6-A0I5-01 | GATA3, MLL3 |
| TCGA-B6-A0I6-01 | ZNF808, H6PD, IL12RB1, UCP2 |
| TCGA-B6-A0I8-01 | IL1R2, NPAS3, OR7A10, SYT8, TEX15, TTN, UGT8, ZBED5, ZCCHC12, COL23A1, FAM98A, TMEM72, C2orf84, NCOR1 |
| TCGA-B6-A0I9-01 | DNASE2, FAM171A1, IVNS1ABP, MPDZ, FAM82B, IGSF9B, NSDHL, OR1M1 |
| TCGA-B6-A0IA-01 | TTN, MMEL1 |
| TCGA-B6-A0IB-01 | C14orf43, POLR3F, SPTBN5, ZFP36L1, COL20A1, MAP2K4, ANKRD11, NLRP1 |
| TCGA-B6-A0IC-01 | ANO8, CEP350, EDC4, OBSCN, ZCCHC6, ZFYVE9, DUSP16 |
| TCGA-B6-A0IE-01 | LOC100287414, VCAN, HPS3, LOC100288182, ZNF439 |
| TCGA-B6-A0IG-01 | NSMCE4A, PHF20 |
| TCGA-B6-A0IH-01 | RFX3, GRB10, RUNX1 |
| TCGA-B6-A0IJ-01 | SIK1, HPD, LDOC1L, LMTK2, MYO10, PLCZ1, PRB3, SDK2, TRPC7 |
| TCGA-B6-A0IK-01 | AGTPBP1, ARID4B, BTN2A2, CNTN2, EML5, GPR85, GPRASP1, KCNT2, MAGEB17, SMAD2, SSPO |
| TCGA-B6-A0IM-01 | BAG4, GPS2, CEP164, GATA3, LOC100287041, MST1P9, MAP3K1 |
| TCGA-B6-A0IN-01 | FANCA, GIPC3, LOC728531, EIF2AK3, BCMO1, ROCK1, SLC20A2, TTN |
| TCGA-B6-A0IO-01 | IPCEF1, COASY, ANAPC5, MAP3K1 |
| TCGA-B6-A0IP-01 | CDH1, MAGIX, ZNF594, PTPRB, AIM1, MAGEA10, MLL3, MYO9A, NCOR1, RETSAT |
| TCGA-B6-A0IQ-01 | CD2BP2, CTNND2, DNAH1, IFITM1, LRRC2, LOC400986, TJP1, TNIP2, DGKI, DOPEY1, NOTCH3, SFRP1, TP53 |
| TCGA-B6-A0RE-01 | LSM4, PHKG2, TP53, EXTL3, MMP17, TAS2R43, APOB, C1orf26, LAPTM4B, SLCO2A1, ZC3HC1, EPS8L1 |
| TCGA-B6-A0RG-01 | CDK18, KIAA0430, LZTR1, MAP2K4, NRK, ZNF852, ARID1A, CFTR, SYNM |
| TCGA-B6-A0RH-01 | CLEC4E, PPP1R14A, IP6K2, LOC100288508, MSLNL, MAP2K4, DEPDC7, DTNB, SI |
| TCGA-B6-A0RI-01 | MAP2K4, ZC3H18, SLC26A4, TBX3, PKN2 |
| TCGA-B6-A0RL-01 | DHRS9, SLC6A18, AKT3, EP300, GBP2 |
| TCGA-B6-A0RM-01 | NAB1, RTN1, TRPS1, KIAA1267, MAP3K1 |
| TCGA-B6-A0RN-01 | JUN |
| TCGA-B6-A0RO-01 | FOLR4, LGI2, GPC1, GRHL2, SPEN, TMEM151B |
| TCGA-B6-A0RP-01 | ENSG00000215099, CASP8 |
| TCGA-B6-A0RQ-01 | DEPDC1, CDH1 |
| TCGA-B6-A0RS-01 | USP12, WDR6, SYN3, TDRD12, TFB1M |
| TCGA-B6-A0RT-01 | KCNH6, NLRP13 |
| TCGA-B6-A0RU-01 | ACSF2, ACTL6B, IGSF5, MED27, SILV, SLC25A13, VPS11, CD248, LOC100289403, TP53, UTRN, ZNF781 |
| TCGA-B6-A0RV-01 | DNALI1, ARID2, JAGN1 |
| TCGA-B6-A0WS-01 | LRBA, GATA3, WDR74 |
| TCGA-B6-A0WT-01 | ARID1B, ALG10 |
| TCGA-B6-A0WV-01 | FAM166A, UBE2A, ZNF292, CCDC159, MLL3, GSPT1 |
| TCGA-B6-A0WW-01 | LCE1D, SNRPB, CASP6, PTEN, TPI1 |
| TCGA-B6-A0WX-01 | NPDC1, MANEA, TP53 |
| TCGA-B6-A0WY-01 | CHD1L, MLL4, NCOR1, NRAP |
| TCGA-B6-A0WZ-01 | C22orf46, FUT2, NEB, ZFYVE16 |
| TCGA-B6-A0X0-01 | GATA3, STAT4 |
| TCGA-B6-A0X1-01 | BRCA1, DST, OR5AK2, TRPA1, USP43, GRIN2A, KRTAP2-1, MASP1, UNC5D, XPO6 |
| TCGA-B6-A0X4-01 | KIAA0182, GRIK2, MLL3, RNF217, ZNF229 |
| TCGA-B6-A0X5-01 | LOC100287244, ENSG00000213926, ENSG00000230264, PHF20L1, SLC13A5, CENPL |
| TCGA-B6-A0X7-01 | AASDH, MLLT4 |
| TCGA-BH-A0AU-01 | C19orf45, HLA-G, PCDH9, GALNT7, ARNT, NMS, ZNF207 |
| TCGA-BH-A0AV-01 | DPRX, RHO, EPN3, NLRC5, PNMAL1 |
| TCGA-BH-A0AW-01 | APOB, ITSN2, KCNK9, TRIM13, WDR49, ZFHX4, ANKAR, DNAJC27, LPO, PDP1, PRAME, RAB11FIP4, SERPINA7, TIAM1, FLJ43860, GTPBP1 |
| TCGA-BH-A0AY-01 | IFT140, PCDHB18, ZNF687, CD74, FUK, PI4KA, BSX, EEF2K, PIWIL1 |
| TCGA-BH-A0AZ-01 | CUBN, PLK2, PRDM15, C22orf33, GATA3, PTPRU |
| TCGA-BH-A0B0-01 | RASGRF1, FBXL13 |
| TCGA-BH-A0B1-01 | MAP7, TFPI2, TPM1, ZBTB17, ARHGEF17, IFNA7 |
| TCGA-BH-A0B3-01 | ADAMTS18, KCNT1, LPIN2, AFF2 |
| TCGA-BH-A0B5-01 | GRK1, CDKN1B, CHPT1, CNTN6, NRBP2, SSPO |
| TCGA-BH-A0B7-01 | TPST1, WNT7A |
| TCGA-BH-A0B8-01 | SPEN, AIM1L, CBLN1, SMARCA4, ANXA13, POP5 |
| TCGA-BH-A0BA-01 | PCDHB6, SAAL1, VILL, MSLNL, PHF12 |
| TCGA-BH-A0BC-01 | PLA2G4D, AHCTF1, MEF2C, TRAT1, ZNF415 |
| TCGA-BH-A0BD-01 | UBC |
| TCGA-BH-A0BF-01 | PTGER1 |
| TCGA-BH-A0BG-01 | LOC100130814, TP53, LOC134505, LOC284395, TRAPPC10 |
| TCGA-BH-A0BJ-01 | C19orf57, LOC100286994, PTCHD1 |
| TCGA-BH-A0BL-01 | CNOT3, HMHA1, SLC39A14, DLX5, FBXO44, GOLGB1, RGPD4, SLC25A25 |
| TCGA-BH-A0BM-01 | CACNA1B, DDX5, LOC100289633, RUNX1, KRT36, RAD54B |
| TCGA-BH-A0BO-01 | LIFR, MAP3K1, HTT |
| TCGA-BH-A0BP-01 | HPSE, MAP3K4, MRC2, ZNF717, DBNDD2, C9orf41, CCDC113, CHD3, LOC643677, VSIG10 |
| TCGA-BH-A0BQ-01 | C2orf86, DSG3, PKNOX2 |
| TCGA-BH-A0BR-01 | RNF14, GATA3, ROBLD3, C4orf40 |
| TCGA-BH-A0BS-01 | PDCD6IP, GATA3, CCDC66, KRT38 |
| TCGA-BH-A0BT-01 | NDUFS7, FAM175B |
| TCGA-BH-A0BV-01 | COX10, ABCB11 |
| TCGA-BH-A0BW-01 | AGPAT5, ALDH8A1, BCL9, EIF2C3, ENSG00000173213, MED12, PNPLA3, SARM1, ZNF730, RB1, ACRBP, BAZ1A, FRAS1, LOC100288413, POLR1C |
| TCGA-BH-A0BZ-01 | CCDC121, CD5, EHD3, KRT82, ST8SIA6, SYNE1, APP, PPAPDC1B, ZZEF1, KIF20B, ZNF552, ANKMY1, BRE, C20orf152, CDV3, HEG1, ITM2A, KCTD21, MAN1A2, OR52N2, SYMPK, TLR4, TMEM131, TMEM201 |
| TCGA-BH-A0C0-01 | LOC100287962, TP53, NRP2, CAMK2D, RGS4 |
| TCGA-BH-A0C1-01 | BAZ2A, CDH1, COL17A1 |
| TCGA-BH-A0C3-01 | NHS, AMELX, FUT2 |
| TCGA-BH-A0C7-01 | DDX43, GATA3, RB1, KCNB2, KCNMB2, OBSCN |
| TCGA-BH-A0DD-01 | E2F3, NOSTRIN, RAB2A, FREM3, GZMM |
| TCGA-BH-A0DE-01 | RPL5, MLL3, GRIN2A |
| TCGA-BH-A0DG-01 | GATA3 |
| TCGA-BH-A0DH-01 | LPA |
| TCGA-BH-A0DI-01 | GPSM2, ZNF93 |
| TCGA-BH-A0DK-01 | ANKRD12, FHDC1, POL3S, ANKRD11, GATA3, MALAT1, PHTF2, C6orf136, DOCK7, ILF2, KIAA1524, NGFR, SLC25A16 |
| TCGA-BH-A0DL-01 | KDM6A |
| TCGA-BH-A0DO-01 | EGR3, HIVEP2, MMP12 |
| TCGA-BH-A0DP-01 | LOC100132364, MTMR15, KY, NPY1R |
| TCGA-BH-A0DQ-01 | KIAA0913, MAP2K7, TECTA, TSGA10 |
| TCGA-BH-A0DS-01 | HOMEZ, STAU2, GATA3, TRPM8, ZNF644 |
| TCGA-BH-A0DT-01 | BMPR1B, GATA3, LOC100287102, MAP3K1 |
| TCGA-BH-A0DV-01 | MLL3 |
| TCGA-BH-A0DX-01 | MED24, ENSG00000154898, ITGB2, KIAA1012, PPIL5, TBC1D9 |
| TCGA-BH-A0E0-01 | ACE, ALK, LAMA1, RABGAP1 |
| TCGA-BH-A0E1-01 | LOC100289661, LOC284288, PRPF40B, TAS2R10 |
| TCGA-BH-A0E2-01 | FANCM, MLLT10 |
| TCGA-BH-A0E6-01 | LRP12, NOTCH2, TP53, ASXL2, GALNT7, NEK9 |
| TCGA-BH-A0E7-01 | MAP3K1, TBX3, ZMYM3, MTMR9, C14orf39, HIRA, SCYL1, TLR2 |
| TCGA-BH-A0E9-01 | RBM5, PTEN, CDH1, INTS4 |
| TCGA-BH-A0EA-01 | GATA3, RTTN, TXNDC15 |
| TCGA-BH-A0EB-01 | SENP1, CHRNB2, MLLT4 |
| TCGA-BH-A0EE-01 | ARCN1, GALNT4, ERGIC1, CNKSR2, CUL4B, COX10 |
| TCGA-BH-A0EI-01 | DSPP |
| TCGA-BH-A0GY-01 | AGL, CARM1, FBXO41, LPHN1, NEB, SLC27A5, GRLF1 |
| TCGA-BH-A0GZ-01 | TRMT61B, PTEN, YLPM1 |
| TCGA-BH-A0H0-01 | EZR |
| TCGA-BH-A0H3-01 | MUC6, GATA3, ATP1B1, NCOR1 |
| TCGA-BH-A0H6-01 | EWSR1, MAP3K1, CHGB |
| TCGA-BH-A0H7-01 | CCL4L2, CHD6, XPNPEP3 |
| TCGA-BH-A0H9-01 | CA11, COL11A2, PPP1R9A, MAOA, LMOD3, PLCL2 |
| TCGA-BH-A0HA-01 | ARHGAP29, DALRD3, ENSG00000214982, FAM35B, MED23, POLR2B, SLTM, VPS4A, TRIM50, AP4M1, EPAS1, GRM3, INTS8, MLH1, MRGPRX1, ODZ3, OSBPL1A, RAB11A, TRIM32, ZNF645 |
| TCGA-BH-A0HB-01 | LEPR, LRBA |
| TCGA-BH-A0HI-01 | GINS2 |
| TCGA-BH-A0HK-01 | HIST1H3B, RASSF5, GTPBP8, COL3A1, FAT4, TINAG |
| TCGA-BH-A0HO-01 | BACH1, ENTHD1 |
| TCGA-BH-A0HP-01 | FAM166A, PREX1, RENBP, TRIP12, RUNX1, CHD1L, CYP2C18, GDPD2, MDC1, NEK5, PTPRH, SPEN, SPRYD5, ZHX1, ZNF283, ZNF385B |
| TCGA-BH-A0HQ-01 | AQP7, MLL3, MYB, RUNX1, ZFHX3 |
| TCGA-BH-A0HU-01 | CYP4V2, TMEM30B |
| TCGA-BH-A0HW-01 | KHK, LOC100128827, LOC400352, LRP3, NODAL, SLC9A3, WHSC2, CEP55, ZNF135, ARID1A, KRT81 |
| TCGA-BH-A0HX-01 | FAM171A1, AP2A1 |
| TCGA-BH-A0HY-01 | CGN, ENSG00000180923, SLC4A1AP, TBC1D5 |
| TCGA-BH-A0RX-01 | FAM117B, FAM53C, MUC6, TP53 |
| TCGA-BH-A0W3-01 | COQ9, LOC100291056 |
| TCGA-BH-A0W4-01 | CDKN1B, ZNF592, KIAA1033, PCDH15 |
| TCGA-BH-A0W5-01 | MAP3K1 |
| TCGA-BH-A0W7-01 | LOC100288105, ADAM29, C10orf18, C12orf64, GRIK1, HSF5, LARS2, MAP2K4, NRK, PCDHGB2, VPRBP |
| TCGA-BH-A0WA-01 | FBXW4, GCC2, KIAA0467, KISS1, PRUNE, ZNF619 |
| TCGA-BH-A18F-01 | AP4M1, NF1, CDH1, SEC31A, GTF2IRD2 |
| TCGA-BH-A18H-01 | NDUFAF1, LOC441806, SPEN, TP53 |
| TCGA-BH-A18I-01 | HMBOX1, LCT, BCHE |
| TCGA-BH-A18J-01 | MYO10, ABCD2, EIF1AX, KLRAQ1, PAN2, SEMA5A, TBC1D24 |
| TCGA-BH-A18K-01 | EPB41L4A, SOX9 |
| TCGA-BH-A18L-01 | AIDA, FAM169A, JMJD1C |
| TCGA-BH-A18M-01 | CDCP1, MAP3K1 |
| TCGA-BH-A18N-01 | MLL3, SLC9A4, MAP3K1, PCDHGB6 |
| TCGA-BH-A18P-01 | CDH1, INO80, MAP3K1, TBL1XR1, AS3MT, CAD, CARD6, CSF2RB, DNAJA3, FAP, GLS, MYO7B, NCOA2, NKAP, PARP2, PLCXD3, POLQ, RPAP1, RPAP3, RSPH4A, SH3TC2, SLC17A8, SPN, TLR4, TTN, WDR45L, WDR7, XIRP2, ZNF623 |
| TCGA-BH-A18Q-01 | EPHA3, LOC100287244, MGAT5B, SIN3B, AASS, COL25A1, TP53, SAMD3, ZBTB40, ZIM2 |
| TCGA-BH-A18R-01 | CCDC18, TBL1XR1, PITX2 |
| TCGA-BH-A18S-01 | C2orf65 |
| TCGA-BH-A18T-01 | PIP4K2C |
| TCGA-BH-A18U-01 | CYP7B1, TMCO7, TP53, GJA10, GOLGA3, LCA5L, RBM34, WBSCR17, ZFP3 |
| TCGA-BH-A18V-01 | ACTN4, ARID5B, KIAA0408, OR56A4, MYBPC3, STRA8, BARX2, C14orf106, C2orf81, CLMN, FGF7, GRM8, RGS3, STMN4, TP53 |
| TCGA-BH-A1EO-01 | ASB5, RUFY1, AKT3, ARID1A, C4orf23, CD1D, DET1, ZNF831 |
| TCGA-BH-A1ET-01 | MTPAP |
| TCGA-BH-A1EU-01 | CCR3 |
| TCGA-BH-A1EV-01 | LRBA, RUNX1, ARPP21 |
| TCGA-BH-A1F0-01 | TP53, DCDC5, ELTD1, SGK1, ZDBF2 |
| TCGA-C8-A12K-01 | C13orf3, LTBP2, RPGR, VASN, FAM183B, NUDCD1, PNPO, PSKH1, SMTN, TUT1 |
| TCGA-C8-A12L-01 | FLNB, PGLYRP2, DMGDH, SFI1, TAF1L, ZNF295 |
| TCGA-C8-A12M-01 | MYO6, PSTK, SMURF2, GATA3, RASA1, KIAA1407, NEIL1, PTPRB, PTPRQ |
| TCGA-C8-A12N-01 | MAP3K1, MLL2 |
| TCGA-C8-A12O-01 | DNAH17 |
| TCGA-C8-A12P-01 | CHERP, FAM111B, PKHD1L1, SETD2, BRAF, ENSG00000212664, PCDHGA6, PDIK1L, TBL1X, UBAP2, ATP13A1 |
| TCGA-C8-A12Q-01 | XBP1, GZMK, BAZ2B, MOAP1, NFE2L1, PEG3, SLC2A11, ZFP106, ZNF280B, ZPLD1 |
| TCGA-C8-A12T-01 | STARD9, AP4E1, ARID1A, CASK, CTCF, GK2, MUC17, PIGQ, TNPO2, UBR3, ZFHX3, ZNF841 |
| TCGA-C8-A12U-01 | ASH1L, AKR1B1, ALDH1L2, MAGI3, MAP3K6 |
| TCGA-C8-A12V-01 | OPN4 |
| TCGA-C8-A12W-01 | WDR78 |
| TCGA-C8-A12Y-01 | PLEKHF2, SLC35D2, DSC3 |
| TCGA-C8-A12Z-01 | TRIP10, RGS22 |
| TCGA-C8-A130-01 | TP53, METAP2 |
| TCGA-C8-A131-01 | ACTN2, GRPEL2, MYH7, TBX3, TEP1 |
| TCGA-C8-A132-01 | PPP1R3A, RGMB, ERCC1, LRRK2 |
| TCGA-C8-A133-01 | GATA3, LOC653513, RMND1 |
| TCGA-C8-A134-01 | H2BFWT, PPP1R3A, AIG1, FARP2, NAV2, SLC25A45 |
| TCGA-C8-A135-01 | RABGEF1, TP53, SVEP1, EML4, AKR1C2 |
| TCGA-C8-A137-01 | PAX5, HRNR, LOC653061, DCST1, MAZ, ZNF498, SERPINA6 |
| TCGA-C8-A138-01 | TP53, TRAPPC10, BRMS1, RARB, SLC22A3 |
| TCGA-C8-A1HF-01 | ARMC8, ENSG00000237362, IFIT5 |
| TCGA-C8-A1HG-01 | ATP9A, DPY19L2, SEMA3D, SSH1 |
| TCGA-C8-A1HI-01 | TECPR2, GATA3 |
| TCGA-C8-A1HL-01 | ARID1A, GATA3, PELI3, TRPM3 |
| TCGA-C8-A1HM-01 | ZNF638, CLIC5, LOC100288571, AKAP11, BCO2, FARP1, FREM3, GCN1L1, GRIA3, KANK2, KCNT2, KNTC1, LRP1, MAPRE3, OR52E4, PCLO, PDCD11, RPE65, SLCO1B1, SNRNP35, TMEM229A, UNC13C, ZFPM2 |
| TCGA-C8-A1HN-01 | KIF14, WDR72, GATA3, TBX3, KCNJ3, SEMA3F, VWF, ZFP36L1 |
| TCGA-D8-A13Y-01 | C2orf51, CSPG4, KIAA1755, OR52H1, TP53, MED8, TBKBP1 |
| TCGA-D8-A13Z-01 | KIAA0141, PTEN, FYCO1, DDX39, MAP3K13, TARBP2 |
| TCGA-D8-A140-01 | RAN, C15orf52, FAM166A, OBSCN, CXorf57, FER1L5, ITPR1, KCNK13, PPAPDC1A, SEC13, ZNF347, ZP4 |
| TCGA-D8-A141-01 | NEK3 |
| TCGA-D8-A142-01 | LOC342346, MUC5B, ETF1, FAM58A, MYOT |
| TCGA-D8-A143-01 | MUC12, FAM40A, RB1, BMP2K, GPR133, OBSCN |
| TCGA-D8-A145-01 | CEP170, WWC3, LOC645605 |
| TCGA-D8-A147-01 | FAM188B, LATS1, PKHD1L1, SPEG, C6orf208, A4GALT, DMGDH, FRMD7, TRIM6-TRIM34, WFDC6 |
| TCGA-E2-A105-01 | MEN1, NCOR1, SP3, ZNF804B, FAM111A, MBD6, SPEN, DDX11L8, KIAA1409, PDE4DIP, TLR8 |
| TCGA-E2-A108-01 | C1orf124, CCDC34, CD40, ZNF695 |
| TCGA-E2-A109-01 | C1orf226, CHD6, CHORDC1, ELP3, IFT74, MLLT4, SDR39U1, SPATA4, STAC, ZFYVE9, FOXP3, ZNF512, KCP, KLF15 |
| TCGA-E2-A10A-01 | ESR1, GATA3, MAP3K1, LTBP4 |
| TCGA-E2-A10B-01 | WDFY3, AADAC, PCDH7 |
| TCGA-E2-A10C-01 | FAM193A, ZNF142, ABCD1, ADAM33, BMP2, C11orf30, CAPN13, CHD6, DCTN1, FAM120A, FANCD2, GDPD2, KCNT2, KRT38, LIFR, METTL13, NUFIP2, NUP205, OR2F1, OR6A2, RGS4, SCN3A, SCYL3, SLC28A2, SLC32A1, THSD7A, TUBA3C, TYW1B, VCL, ACBD5, OR6C2 |
| TCGA-E2-A10E-01 | TG |
| TCGA-E2-A10F-01 | CDH1, NRK, STAG1 |
| TCGA-E2-A14N-01 | SYCP2L, THNSL1, TP53 |
| TCGA-E2-A14O-01 | ENSG00000220161, LATS1, PJA1, ZNF705G |
| TCGA-E2-A14P-01 | POTEF, HNRNPL, MBOAT4, MCM7 |
| TCGA-E2-A14Q-01 | EML5 |
| TCGA-E2-A14R-01 | PRPF38B, SLC6A1, NBPF12, C7orf60, CROCC, DHX30, GALNTL5, GREB1L, LOC100292564 |
| TCGA-E2-A14S-01 | MAGEA3, PCDH11X |
| TCGA-E2-A14T-01 | NUDT8, TBC1D4, UBR7, GATA3, HAPLN2, LOC100287684, PTMS |
| TCGA-E2-A14V-01 | OMD, SMG1, AMAC1L1, DISP1, ITLN2 |
| TCGA-E2-A14W-01 | ADAMTS1, BRCA2, GPRIN3, INADL, JAK3, KCNH7, MYPN, OR7D4, PLEKHA8, SLC24A6, ZFYVE16, SHROOM4, ATP13A2, C17orf95, SUMO1 |
| TCGA-E2-A14X-01 | TP53 |
| TCGA-E2-A14Y-01 | MGA, C11orf85, LOC732391, PCDH15 |
| TCGA-E2-A14Z-01 | PTEN, CCNB3, GSTA4, RASA2 |
| TCGA-E2-A150-01 | C10orf47, BCAS1, DNAH9, NCCRP1, RB1CC1, TP53 |
| TCGA-E2-A152-01 | HIST1H2BC, HMGN5, MAP4, PPM1E, SNX7, SPARCL1, SPINT3, SPRR2F, TEAD1, TTC18, UBE2V1, UNC13C, ZNF469, ZNF480 |
| TCGA-E2-A153-01 | CBFB, TRMT5 |
| TCGA-E2-A154-01 | ALS2CR11, RUNX1, DUPD1, ENSG00000185864 |
| TCGA-E2-A155-01 | AKAP9, ATIC, NYX, GATA3, CD1A, NLRC4, USP36, ZNF597, ZNF646 |
| TCGA-E2-A158-01 | FLT3LG, CNTNAP2, IGSF1 |
| TCGA-E2-A159-01 | RB1, C20orf26, CENPO, ENSG00000197309, IVD, LAMB1, NPAT, PCTK1, RBM14, STAG3 |
| TCGA-E2-A15A-01 | ZNF10, PHF16, FBXL17, PTPRD, SORCS1 |
| TCGA-E2-A15C-01 | MAP3K1, TTC21B |
| TCGA-E2-A15D-01 | LOC100289538, CD69, MAP1A |
| TCGA-E2-A15G-01 | MAP3K1, ZFYVE27, HMCN1, C6, CCDC67, GDF6, MYB, TBL1XR1 |
| TCGA-E2-A15H-01 | DMP1, GATA3, FGD4 |
| TCGA-E2-A15I-01 | CHRND, LPO |
| TCGA-E2-A15J-01 | GATA3, BET1L, PYCARD, VGLL4 |
| TCGA-E2-A15K-01 | NFATC4, PIWIL2, MLLT10, ZNF675 |
| TCGA-E2-A15L-01 | ACE2, PRICKLE2 |
| TCGA-E2-A15M-01 | ATG4A, DCT, DMD, IFI35, KLRC1, ENSG00000214210, LAMA5 |
